# UBXD1 is a mitochondrial recruitment factor for p97/VCP and promotes mitophagy

**DOI:** 10.1038/s41598-018-30963-z

**Published:** 2018-08-17

**Authors:** Ana C. Bento, Claudia C. Bippes, Corina Kohler, Charles Hemion, Stephan Frank, Albert Neutzner

**Affiliations:** 1 Department of Biomedicine, University Hospital Basel and University of Basel, Basel, Switzerland; 2 Department of Pathology, University Hospital Basel, University of Basel, Basel, Switzerland; 3 Department of Ophthalmology, University Hospital Basel, University of Basel, Basel, Switzerland

## Abstract

Clearance of damaged mitochondria through mitophagy is critical for maintaining mitochondrial fidelity and the prevention of neurodegeneration. Here, we report on the UBX domain-containing, p97/VCP cofactor UBXD1/UBXN6/UBXDC2 and its role in mitophagy. Recognizing depolarized mitochondria via its C-terminal UBX domain, UBXD1 translocates to mitochondria in a Parkin-dependent manner. During Parkin-independent mitophagy, UBXD1 shows no mitochondrial translocation. Once translocated, UBXD1 recruits p97 to mitochondria via a bipartite binding motif consisting of its N-terminal VIM and PUB domains. Recruitment of p97 by UBXD1 only depends on the presence of UBXD1 on mitochondria without the need for further mitochondrial signals. Following translocation of UBXD1 to CCCP-depolarized mitochondria and p97 recruitment, formation of LC3-positive autolysosomes is strongly enhanced and autophagic degradation of mitochondria is significantly accelerated. Diminished levels of UBXD1 negatively impact mitophagic flux in Parkin-expressing cells after CCCP treatment. Thus, our data supports a model, whereby the p97 cofactor UBXD1 promotes Parkin-dependent mitophagy by specifically recognizing damaged mitochondria undergoing autophagic clearance.

## Introduction

Proper mitochondrial function is essential for organismal health, with mitochondrial dysfunction being connected to aging^1^, neuronal cell death and associated neurodegenerative diseases^2^. Multi-tiered machinery is in place to remove and degrade superfluous or damaged proteins to maintain mitochondrial proteostasis^3–5^, cull mitochondrial subunits beyond repair^6^, or remove entire mitochondrial networks through apoptosis^7^. Severe damage to mitochondria exceeding the repair capacity of proteolytic systems, but below the apoptotic threshold, leads to the removal of mitochondrial units through mitophagy^8^. Mitophagic degradation of mitochondria, e.g. following depolarization, is triggered by the stabilization of PTEN-induced putative kinase 1 (PINK1) on mitochondria and the subsequent recruitment of the in-between-ring ubiquitin ligase Parkin. Upon PINK1-mediated phosphorylation of ubiquitin^9^, Parkin is activated and ubiquitinates outer mitochondrial membrane (OMM) proteins leading to the recruitment of autophagy receptors^10^. Among these receptor proteins recruited during mitophagy is the AAA-ATPase valosin containing protein (VCP)/p97^11^. As ubiquitously expressed protein, p97 acts in a plethora of cellular functions involving ubiquitination, including cell cycle control, transcriptional regulation as well as proteostasis. In addition, p97 was recently connected to ubiquitin-mediated degradation of mitochondrial proteins during OMM-associated degradation (OMMAD) and Parkin-dependent mitophagy^12,13^. These multiple diverse functions of p97 suggest tight spatial and temporal control of its activity which is brought upon by the interaction with various cofactors promoting substrate recognition and processing by p97^14^. One of these co-factors is the UBXD1/UBXN6/UBXDC2 protein. Previously identified to be a p97 co-factor^15^, UBXD1 contains from the N- to the C-terminus a valosin-containing protein (VCP) interacting motif (VIM), a peptide N-glycosidase/ubiquitin-associated (PUB), as well as an ubiquitin regulatory X (UBX) domain. Unlike other UBX domain containing proteins which bind to p97 via their UBX domain, UBXD1 interacts with p97 via its VIM and PUB domain^16^. UBXD1 was previously shown to be involved in vesicle trafficking^17^, endolysosomal trafficking^18,19^, autophagic removal of damaged lysosomes (lysophagy^20^), and most recently to OMMAD^21^. Furthermore, UBXD1 is of clinical relevance, as p97 mutations linked to inclusion body myopathy with Paget’s disease and frontotemporal dementia (IBMPFD) and amyotrophic lateral sclerosis (ALS) are defective at interacting with UBXD1^19^.

Here we demonstrate that UBXD1 is involved in p97-dependent steps of mitophagy. We find UBXD1 to localize to mitochondria during carbonyl cyanide *m*-chlorophenyl hydrazine (CCCP)-induced and Parkin-dependent mitophagy. This mitochondrial recruitment is exclusively mediated by the UBX domain of UBXD1. Once translocated to depolarized, Parkin-containing mitochondria, UBXD1 mediates mitochondrial recruitment of p97 in a VIM and PUB domain dependent manner. Furthermore, mitochondrial UBXD1 and p97 induce elevated levels of mitochondrial LC3 and accelerate CCCP-induced and Parkin-dependent mitophagy, while diminished levels of UBXD1 blunt mitophagic flux. Thus, we propose a pro-mitophagic function for UBXD1, which acts as a mitochondrial recruitment factor for p97 during Parkin-dependent autophagic removal of damaged mitochondria.

## Results

**Figure 1:**
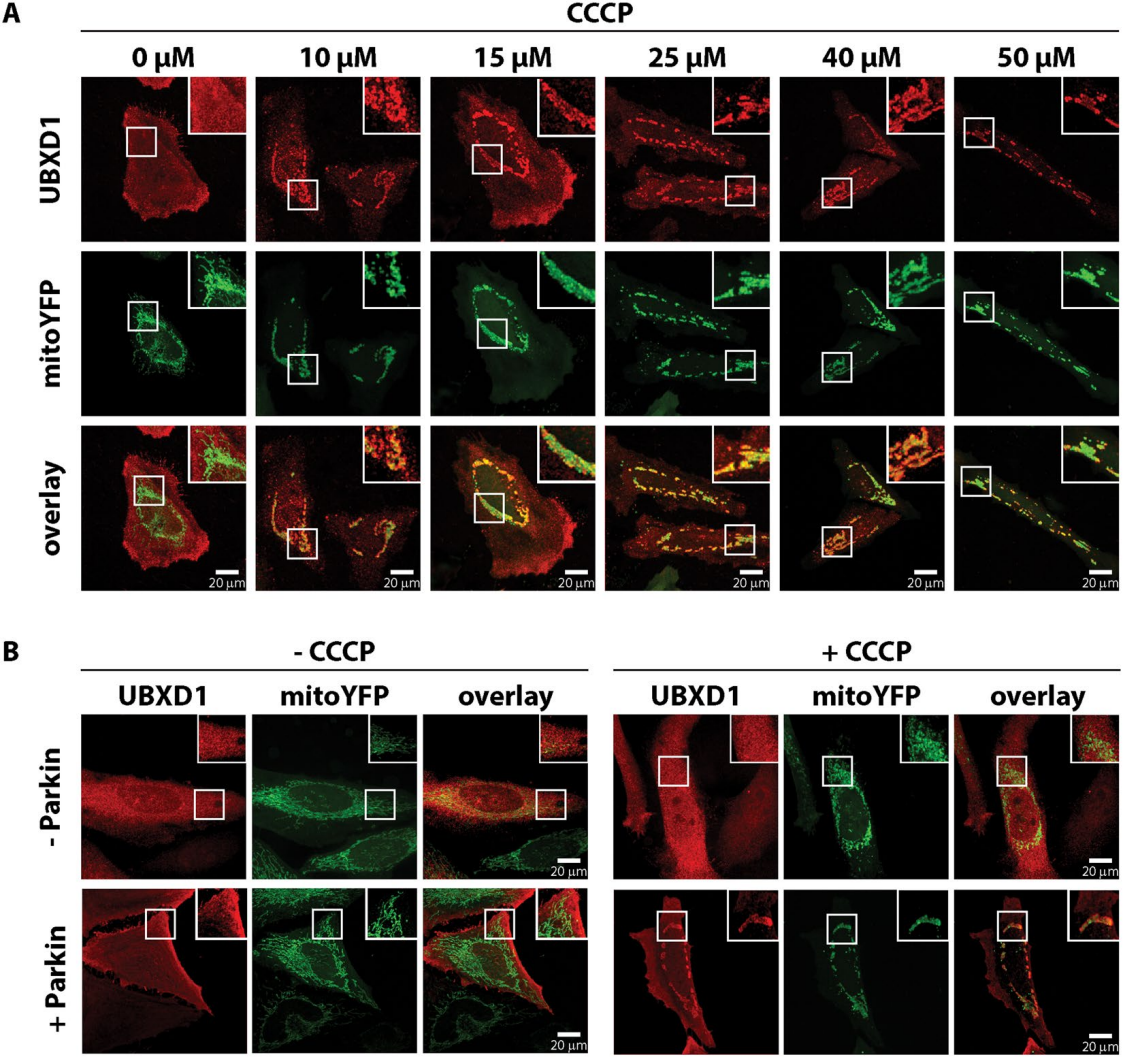
UBXD1 translocates to depolarized mitochondria in a Parkin-dependent manner. (**A**) HeLa cells transfected with expression constructs for FLAG-UBXD1, mitoYFP, and Parkin were treated with 10, 15, 25, 40, or 50 µM CCCP for 6 hours or left untreated as control. Cells were fixed, stained using mouse anti-FLAG antibodies to detect UBXD1 and analyzed by confocal microscopy. (**B**) HeLa cells transfected with expression constructs for FLAG-UBXD1, mitoYFP, and Parkin or vector control were treated with 50 µM CCCP for six hours of left untreated as controls. Cells were analyzed as above.

**Figure 2:**
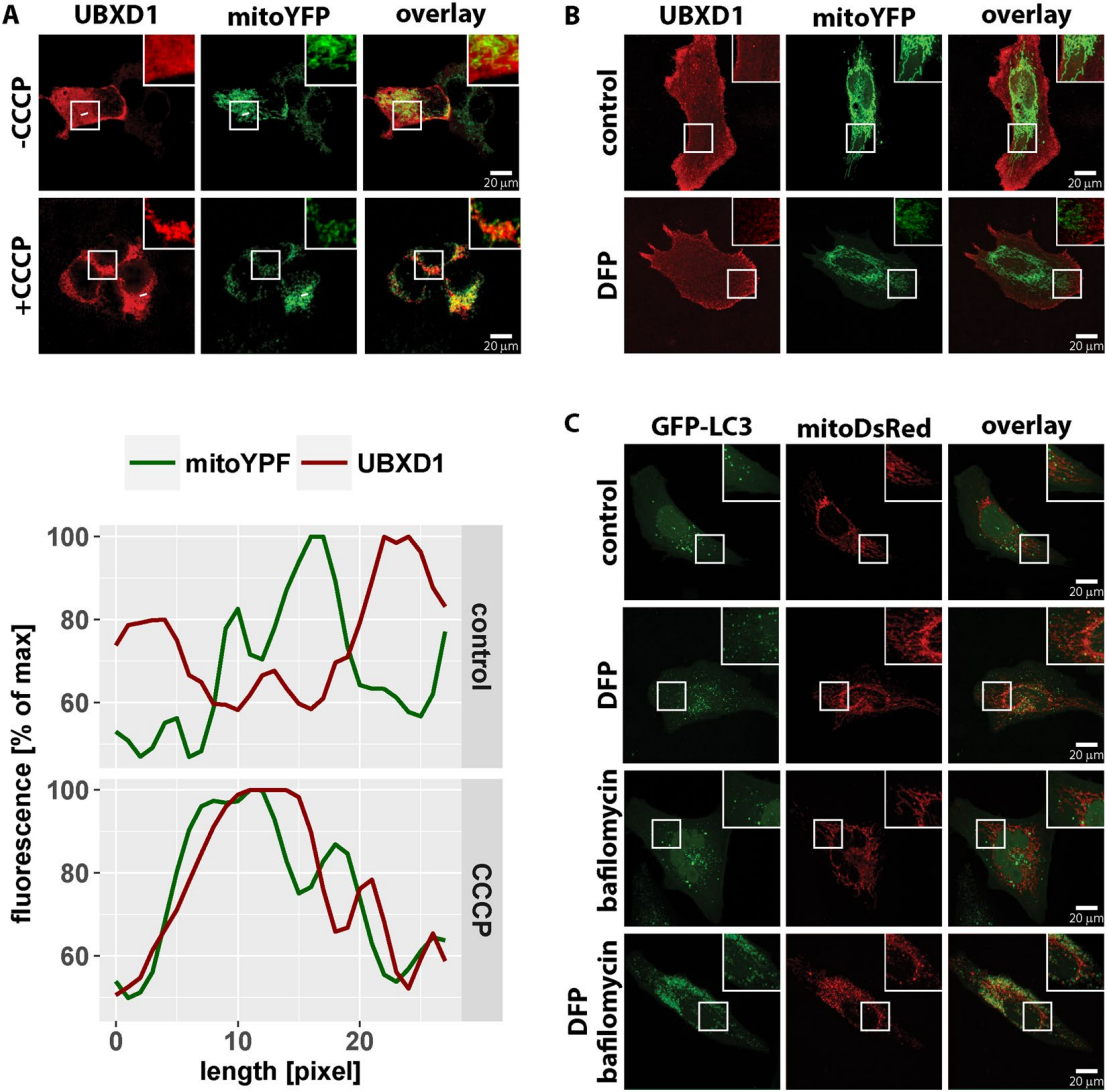
Mitochondrial translocation of UBXD1 in HEK293 cells and during Parkin-independent mitophagy. (**A**) HEK293 cells transfected with expression constructs for FLAG-UBXD1 and mitoYFP were treated for 6 hours with 50 µM CCCP or left untreated as controls. After fixation and anti-FLAG staining, cells were analyzed by confocal microscopy. Fluorescence intensities of FLAG-UBXD1 (red) and mitoYFP (green) along the white line are plotted. Shown are representative images of three independent experiments. (**B**) HeLa cells transfected with expression constructs for FLAG-UBXD1 and mitoYFP were treated for 24 hours with 1 mM DFP or left untreated as control. Cells were fixed, stained using mouse anti-FLAG antibodies to detect UBXD1 and analyzed by confocal microscopy. (**C**) HeLa cells transfected with expression constructs for GFP-LC3 and mitoDsRed were treated for 24 hours with 1 mM DFP, treated for 6 hours with 100 nM bafilomycin or left untreated, fixed and analyzed by confocal microscopy.

As diverse p97 functions are governed by interaction with a plethora of co-factors^14^, and as p97 is involved in the execution of mitophagy^13^, we investigated the mitochondrial translocation of UBX domain-containing p97 co-factors under mitophagic conditions. To this end, HeLa cells co-transfected with expression plasmids for FLAG-tagged UBX domain-containing proteins, mitochondria-targeted yellow fluorescent protein (mitoYFP) and Parkin fused to mcherry (mcherry-Parkin) were treated with the uncoupler carbonyl cyanide m-chlorophenyl hydrazone (CCCP) to induce mitophagy. Upon confocal microscopy analysis of UBX-domain containing p97 co-factors, we identified UBXD1 to translocate to CCCP-uncoupled mitochondria. As shown in Fig. 1A, treatment with increasing CCCP concentrations (0 to 50 µM) of cells expressing mitoYFP-T2A-Parkin-myc3 (see Figure S1) together with FLAG-UBXD1 revealed translocation of UBXD1 at all analyzed CCCP concentrations albeit with increasing efficiency. While UBXD1 showed translocation in about 30% of cells following treatment with 10 µM CCCP, treatment with 50 µM CCCP resulted in mitochondrial translocation in almost all cells. Analyzing induction of apoptosis following treatment with increasing concentrations of CCCP by cytochrome *c* release, we did not find increased cell death after 12 or 24 hours of CCCP treatment regardless of CCCP concentration (see Figure S2A). To assess the Parkin-dependency of mitochondrial translocation of UBXD1, cells expressing FLAG-UBXD1 in the presence or absence of Parkin were treated with CCCP or left untreated. As shown in Fig. 1B, in the absence of Parkin and/or CCCP, Flag-UBXD1 displays cytosolic localization and does not co-localize with the mitochondrial marker mitoYFP. However, in the presence of Parkin and following mitochondrial depolarization, a proportion of UBXD1 translocates to mitochondria. To analyze mitochondrial translocation of UBXD1 in the context of endogenous levels of Parkin, HEK293 cells transfected with FLAG-UBXD1 and mitoYFP expression plasmids were treated with CCCP. As shown in Fig. 2A, treatment with CCCP caused redistribution of UBXD1 from the cytosol to mitochondria. To assess whether UBXD1 is specifically connected to Parkin-dependent mitophagy, mitochondrial translocation of UBXD1 following treatment with the Parkin-independent mitophagic inducer deferiprone (DFP) was analyzed^22^. Treatment of FLAG-UBXD1 expressing cells with 1 mM DFP for 24 hours did not result in redistribution of UBXD1 from the cytosol to mitochondria (Fig. 2B). To assess the extent of mitophagy induced by DFP treatment, HeLa cells expressing mitoDsRed and GFP-LC3 were treated with DFP (24 hours) in the presence of absence of bafilomycin (6 hours) and GFP-LC3 vesicle formation was analyzed. As shown in Fig. 2C, DFP treatment enhanced GFP-LC3 vesicle formation near mitochondria compared to controls cells. This becomes especially evident after additional bafilomycin treatment, where inhibition of lysosomal acidification resulted in the greatly increased accumulation of GFP-LC3 positive vesicles compared to controls. These data are consistent with a specific involvement of UBXD1 in Parkin-dependent but not Parkin-independent mitophagic processes.

**Figure 3:**
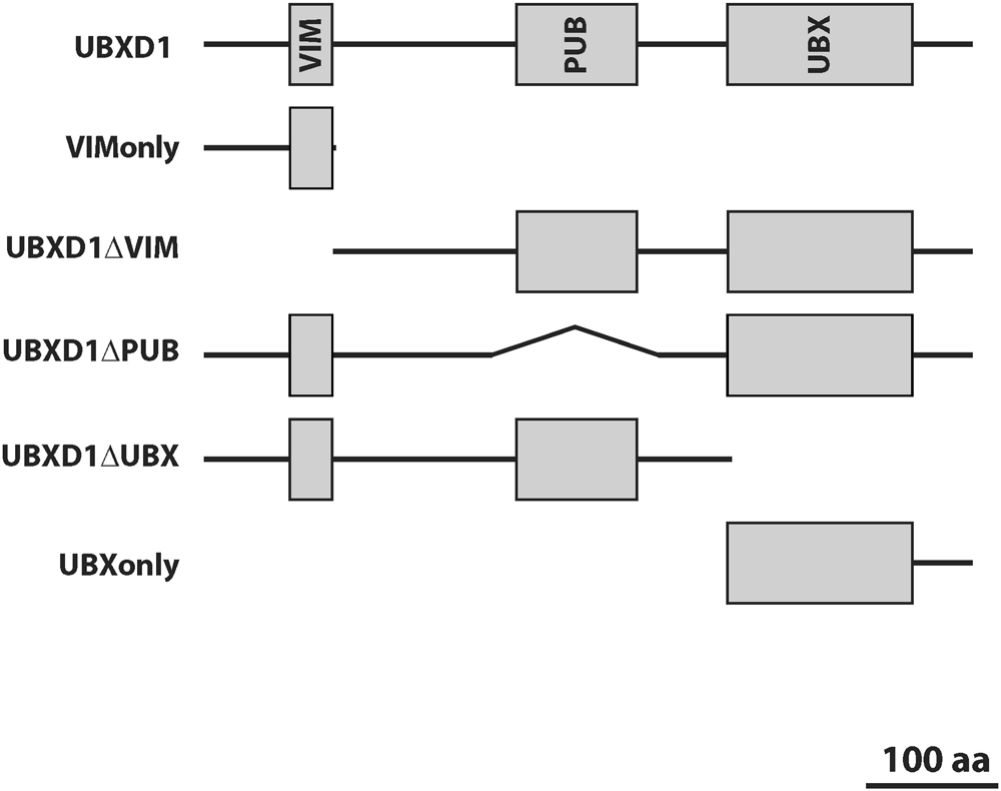
Schematic domain organization of wildtype UBXD1 and mutants used in this study. UBXD1 contains with VIM, PUB and UBX three protein-protein interaction domains. Shown are drawn to scale schematic representation of UBXD1 truncation mutants used in this study.

**Figure 4:**
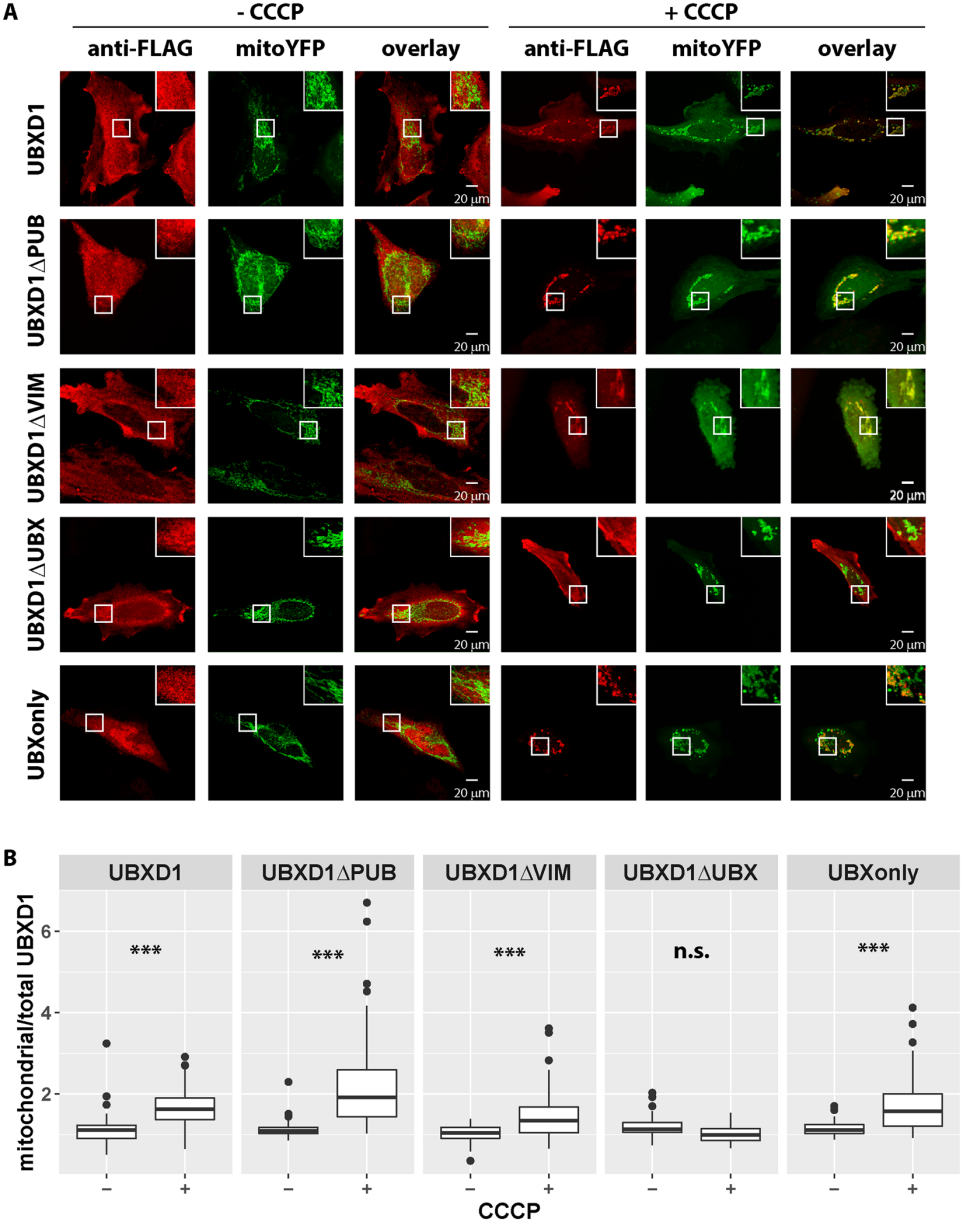
VIM and PUB, but not the UBX domain of UBXD1 are dispensable for mitochondrial translocation during mitophagy. (**A**) HeLa cells transfected with mitoYFP-T2A-Parkin-myc3 and FLAG-UBXD1, FLAG-UBXD1ΔPUB, FLAG-UBXD1ΔVIM, FLAG-UBXD1ΔUBX, or FLAG-UBXonly expression constructs were treated with CCCP for 6 hours or left untreated as control, stained using mouse anti-FLAG antibodies and analyzed by confocal microscopy. (**B**) The ratio of mitochondrial to total FLAG-UBXD1 or various FLAG-UBXD1 variants as measure for mitochondrial translocation was quantified by image analysis of confocal pictures obtain from cells treated as in A. Shown are box plots of three independent experiments with at least 15 cells per experiment and condition. Statistical significance was assessed by ANOVA followed by Student’s t-test using Bonferroni correction to account for multiple comparisons. *** denotes p-values < 0.001, n.s. – no significant difference.

UBXD1 is a modular protein with an N-terminal VIM, a PUB and a C-terminal UBX domain (Fig. 3). To analyze the mitochondrial translocation of UBXD1 under mitophagic conditions (CCCP-mediated mitochondrial depolarization in Parkin expressing cells), FLAG-tagged UBXD1 mutants lacking the VIM (UBXD1ΔVIM), PUB (UBXD1ΔPUB), UBX (UBXD1ΔUBX), PUB and UBX (VIMonly) or VIM and PUB domain (UBXonly) were generated (Fig. 3). HeLa cells transiently co-expressing FLAG-UBXD1 or mutants of FLAG-UBXD1 together with mitoYFP-T2A-Parkin-myc3 were treated with CCCP or left untreated. As shown in Fig. 4A, FLAG-UBXD1 lacking VIM, PUB or both domains were still capable of mitochondrial translocation, similar to wildtype FLAG-UBXD1. Strikingly, FLAG-UBXD1 without the UBX domain lacked mitochondrial localization even under mitophagic conditions. To quantify UBXD1 mitochondrial translocation, the ratio of FLAG-UBXD1 on mitochondria to non-mitochondrial FLAG-UBXD1 was determined (Figs 4B and S3). To assess for potential detection bias in this single cell analysis due to different expression levels of the employed UBXD1 constructs, we performed Western blotting and also analyzed the amount of UBXD1 variants on the single cell level (Figure S4A and B). In line with the findings shown above, a significant difference (p < 0.001) in the mitochondrial to total FLAG-UBXD1 ratio (expressed as median +/− median absolute deviation) between control cells and cells treated with CCCP was found for wildtype FLAG-UBXD1 (no CCCP: 1.11 +/− 0.24; plus CCCP: 1.63 +/− 0.4), FLAG-UBXD1ΔPUB (no CCCP: 1.09 +/− 0.1; plus CCCP: 1.92 +/− 0.93), FLAG-UBXD1ΔVIM (no CCCP: 1.04 +/− 0.21; plus CCCP: 1.34 +/− 0.47) and FLAG-UBXonly (no CCCP: 1.12 +/− 0.16; plus CCCP: 1.58 +/− 0.61), but not for FLAG-UBXD1ΔUBX (no CCCP: 1.14 +/− 0.18; plus CCCP: 0.99 +/− 0.22). These data point to an UBX domain-dependent targeting of UBXD1 to mitochondria under mitophagic conditions, while VIM and PUB domains, whether separate or in concert, are not sufficient to mediate mitochondrial translocation.

**Figure 5:**
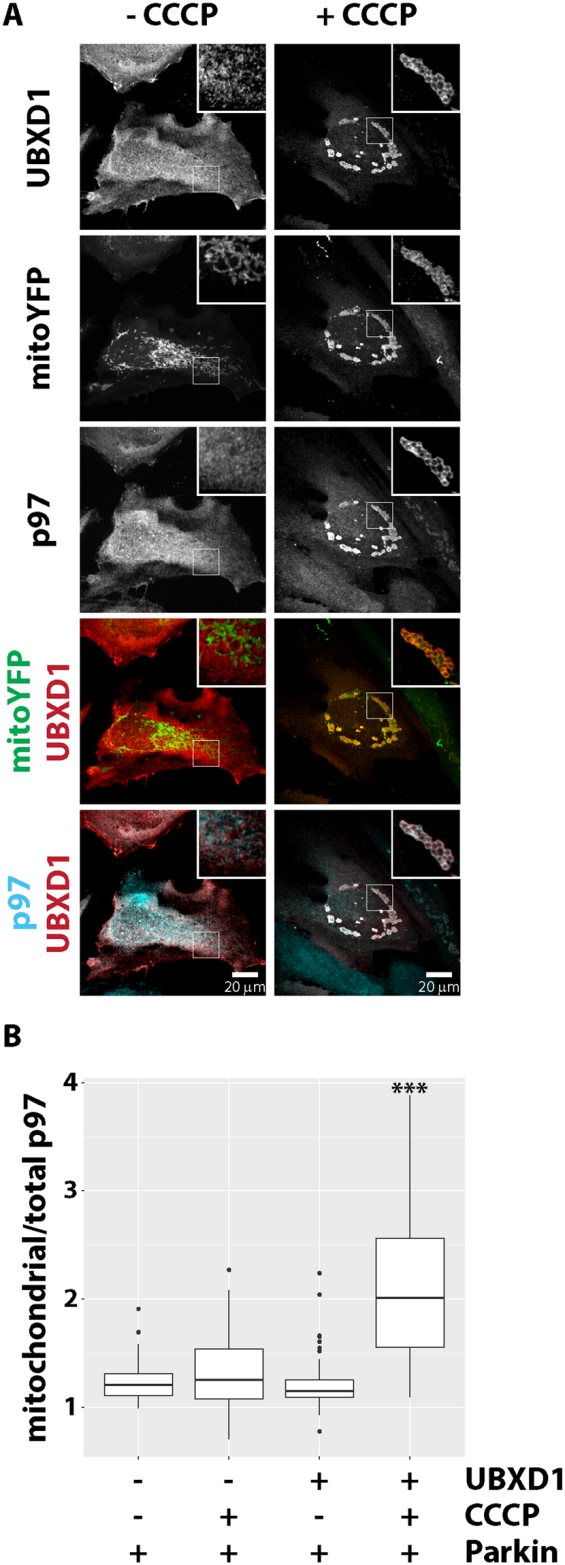
UBXD1 recruits p97 to mitochondria under mitophagic conditions. (**A**) HeLa cells transfected with expression plasmids for FLAG-UBXD1 and mitoYFP-T2A-Parkin-myc3 were treated with CCCP for 6 hours or left untreated as control. Cells were fixed, stained using rabbit anti-FLAG and mouse anti-p97 antibodies and analyzed by confocal microscopy. (**B**) HeLa cells transfected with expression plasmids for FLAG-UBXD1 or vector control and mitoYFP-T2A-Parkin-myc3 were treated as in A. To quantify p97 redistribution to mitochondria, the ratio of mitochondrial p97 to total p97 was determined by image analysis of confocal images. Shown are box plots of three independent experiments with at least 15 cells per experiment and condition. Statistical significance was assessed by ANOVA followed by Student’s t-test using Bonferroni correction to account for multiple comparisons. ***Denotes p-values < 0.001.

As UBXD1 is a known p97 co-factor, we explored the possibility of UBXD1-mediated mitochondrial recruitment of p97 under mitophagic conditions. Analyzing the distribution of endogenous p97 in cells co-expressing FLAG-UBXD1 and mitoYFP-T2A-Parkin-myc3 in the presence or absence of CCCP, we found strong redistribution of p97 from the cytosol to mitochondria upon addition of CCCP compared to controls (Fig. 5A). Quantitative image analysis (Fig. 5B) confirmed a significant (p < 0.0001) accumulation of endogenous p97 on mitochondria in cells with ectopic expression of FLAG-UBXD1 under mitophagic conditions (plus FLAG-UBXD1/plus CCCP: 2.01 +/− 0.7) compared to control cells either with undisturbed mitochondrial membrane potential (plus FLAG-UBXD1/no CCCP: 1.15 +/− 0.24) or lacking ectopic UBXD1 expression (no FLAG-UBXD1/plus CCCP: 1.25 +/− 0.31; no FLAG-UBXD1/no CCCP: 1.21 +/− 0.18). These data are consistent with UBXD1 acting as p97 mitochondrial recruitment factor under mitophagic conditions.

**Figure 6:**
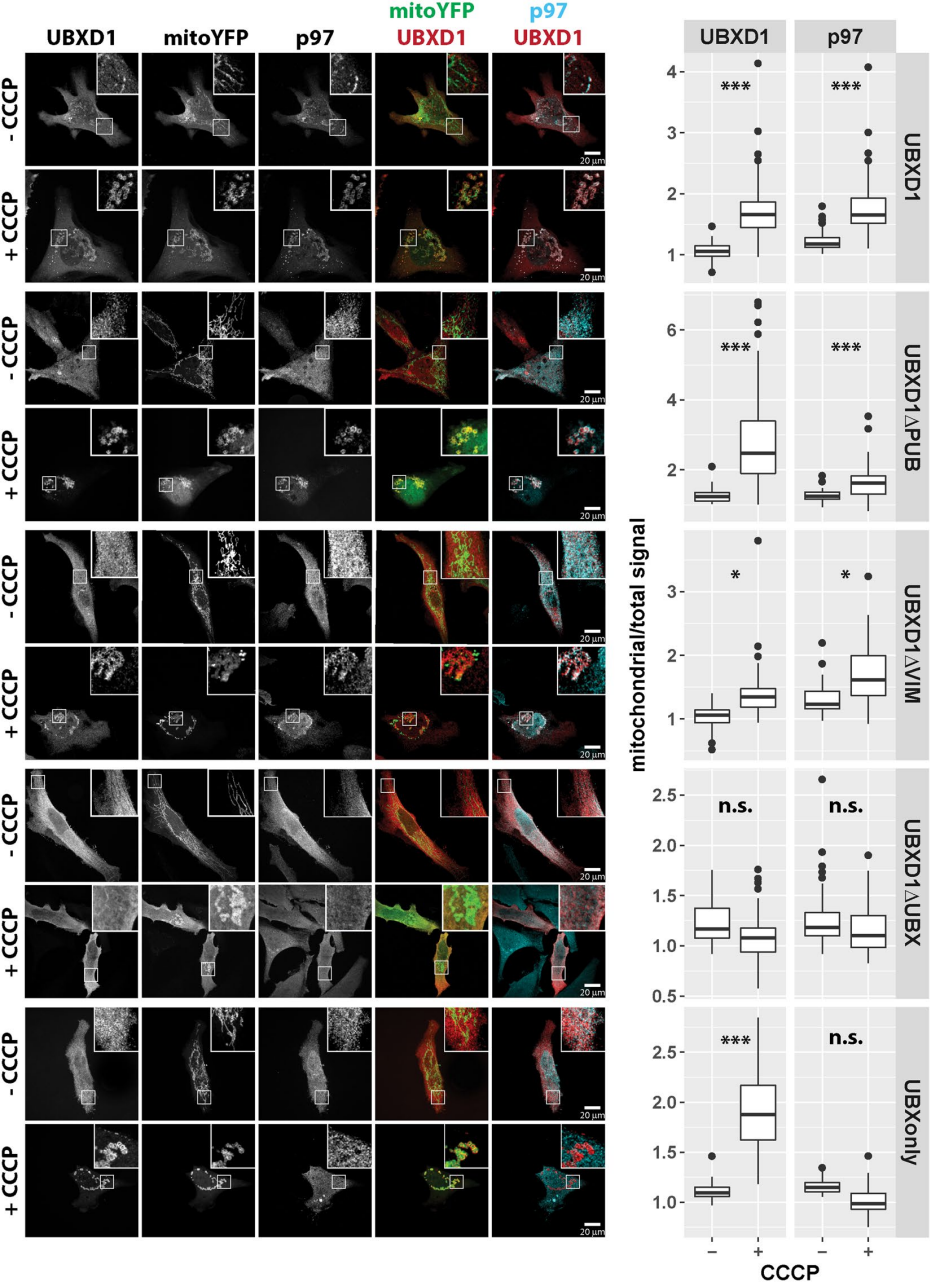
The UBX domain of UBXD1 is essential for mitochondrial translocation of p97. HeLa cells transfected with expression plasmids for FLAG-UBXD1 or variants of FLAG-UBXD1 and mitoYFP-T2A-Parkin-myc3 were treated with CCCP for 6 hours or left untreated as control. Fixed cells were stained using rabbit anti-FLAG and mouse anti-p97 antibodies and analyzed by confocal microscopy. The box plots represent three independent experiments with at least 15 cells/experiment/condition. Statistical significance was assessed by ANOVA followed by Student’s t-test using Bonferroni correction to account for multiple comparisons. *denotes p-values < 0.05, ***p-values < 0.001, n.s. – no significant difference.

To further explore this potential role of UBXD1 in targeting p97 to mitochondria, redistribution of endogenous p97 following expression of FLAG-UBXD1 mutants was analyzed. HeLa cells expressing mitoYFP-T2A-Parkin-myc3 together with wildtype FLAG-UBXD1 or FLAG-UBXD1 mutants were treated with CCCP or left untreated as control, and ratios of mitochondrial p97 to total p97 (p97_m_/p97_t_) and mitochondrial FLAG-UBXD1 to total FLAG-UBXD1 (FLAG-UBXD1_m_/FLAG-UBXD1_t_) were determined (Fig. 6). Confirming our previous findings, FLAG-UBXD1 translocated to mitochondria under mitophagic conditions via its UBX domain, while VIM and PUB domains did not contribute to mitochondrial targeting. As for p97 recruitment, confocal microscopy and determination of p97_m_/p97_t_ revealed significant mitochondrial enrichment of endogenous p97 under mitophagic conditions compared to controls expressing FLAG-UBXD1 (no CCCP: 1.18 +/− 0.14; plus CCCP: 1.65 +/− 0.29), FLAG-UBXD1ΔPUB (no CCCP: 1.25 +/− 0.14; plus CCCP: 1.62 +/− 0.47), and FLAG-UBXD1ΔVIM (no CCCP: 1.23 +/− 0.16; plus CCCP: 1.61 +/− 0.47), but not in FLAG-UBXD1ΔUBX (no CCCP: 1.18 +/− 0.17; plus CCCP: 1.10 +/− 0.21) or FLAG-UBXonly (no CCCP: 1.15 +/− 0.07; plus CCCP: 0.99 +/− 0.1) expressing cells (Fig. 6).

**Figure 7:**
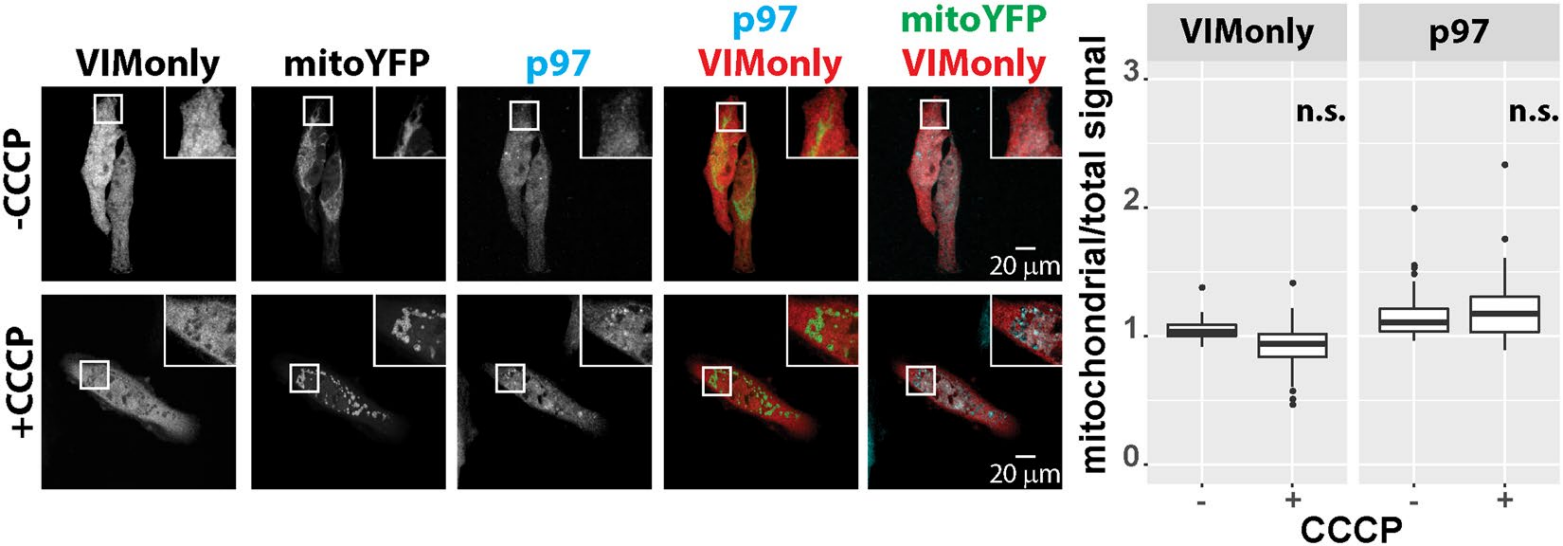
The VIM domain of UBXD1 is not involved in mitochondrial translocation and p97 recruitment. HeLa cells transfected with expression plasmid for FLAG-VIMonly and mitoYFP-T2A-Parkin-myc3 were treated with CCCP for 6 hours or left untreated as control. Fixed cells were stained using rabbit anti-FLAG and mouse anti-p97 antibodies and analyzed by confocal microscopy. The box plots represent three independent experiments with at least 15 cells/experiment/condition. Statistical significance was assessed by ANOVA followed by Student’s t-test using Bonferroni correction to account for multiple comparisons. n.s. – no significant difference.

**Figure 8:**
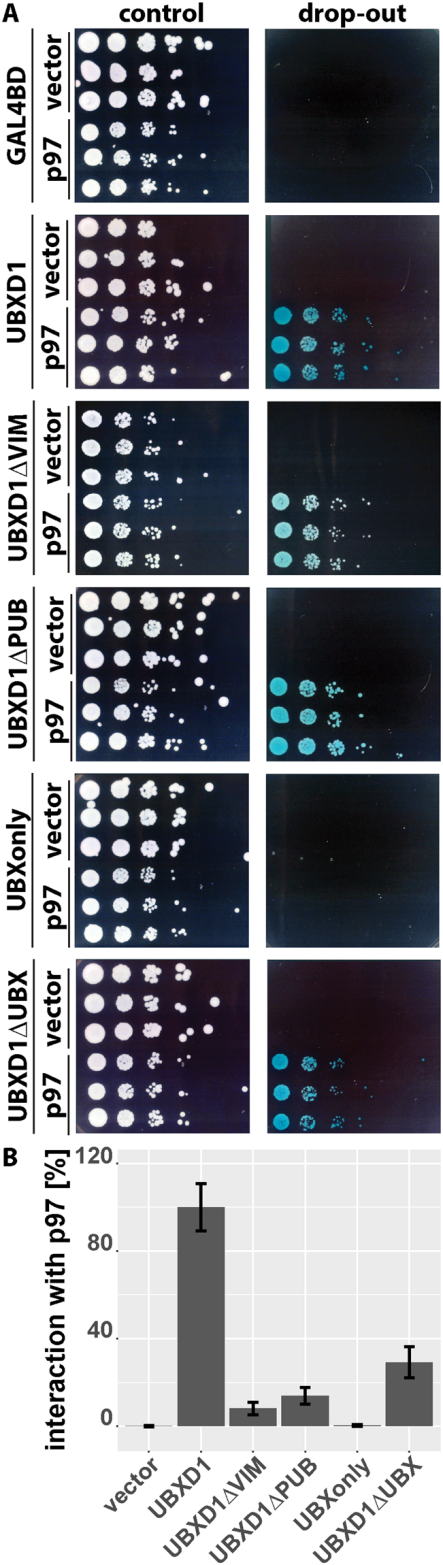
Physical interaction of UBXD1 with p97. (**A**) Cells of yeast strain Y2HGold were transformed with expression constructs for fusion proteins between UBXD1 and the GAL4 DNA binding domain and p97 and the GAL4 activation domain. Transformation with pGADT7 (empty vector with GAL4 activation domain – labeled vector) or pGBKT7 (empty vector with GAL4 DNA binding domain – GAL4BD) served as control. Yeast strains were serially diluted onto plates selecting for expression plasmids (control) and plates selecting for yeast two-hybrid interaction (drop-out). (**B**) Strength of yeast two-hybrid interaction between UBXD1 or variants of UBXD1 and p97 were quantified using a para-nitrophenyl-alpha-galactoside assay. Shown is the average of three independent experiments with five independent yeast transformands per condition. Error bars represent SD.

Analysis of UBXD1ΔVIM (Fig. 4B) revealed significant mitochondrial translocation albeit at somewhat lower levels compared to UBXD1ΔPUB. To address whether the VIM domain might contribute to a certain extend to the translocation of UBXD1, mitochondrial translocation and p97 recruitment activity of the VIM domain (VIMonly) was analyzed. HeLa cells expressing VIMonly and mitoYFP-T2A-Parkin-myc3 treated with CCCP or left untreated showed neither translocation of VIMonly nor of p97 to mitochondria (Fig. 7). So far, these data are consistent with p97 recruitment to mitochondria via the VIM or PUB domain upon binding of the UBX domain of UBXD1 to mitochondria. Thus, a direct physical interaction between the VIM or PUB, but not the UBX domain of UBXD1 and p97 would be expected. To corroborate this potential mechanism, yeast two hybrid analyses were performed. As shown in Fig. 8A, a serial dilution of yeast strains containing Gal4BD-UBXD1 or Gal4BD-UBXD1 mutants as bait and Gal4AD-p97 as prey or GAL4AD vector as prey control on media selective for interaction (drop-out) revealed growth of strains containing Gal4AD-p97 and Gal4BD-UBXD1, or Gal4BD-UBXD1ΔVIM, or Gal4BD-UBXD1ΔPUB or Gal4BD-UBXD1ΔUBX (see Figure S5 for expression control). In contrast, no growth was detected for yeast strains containing empty pGBKT7 bait or Gal4BD-UBXonly. A serial dilution growth assay on media not selective for bait-prey interaction served as control. Our data confirm a direct physical interaction between p97 and UBXD1 via its VIM and also the PUB domain of UBXD1. To elucidate to which extent VIM and PUB domain of UBXD1 contribute to the observed interaction between p97 and UBXD1, we performed a quantitative yeast two-hybrid interaction assay (Fig. 8B). We found no significant difference (p = 0.13) in the interaction strength expressed as percentage of wildtype GAL4BD-UBXD1 between GAL4AD-p97/GAL4BD-UBXD1ΔVIM (8.1 +/− 2.8%) and GAL4AD-p97/GAL4BD-UBXD1ΔPUB (13.8 +/− 3.8%). Interestingly, the interaction strength between GAL4AD-p97 and GAL4BD-UBXD1ΔUBX containing both PUB and VIM domain is 29.2 +/− 7.1% of wildtype GAL4BD-UBXD1. Thus, both VIM and PUB domain seem to contribute about equally to the physical interaction, and without obvious cooperativity.

**Figure 9:**
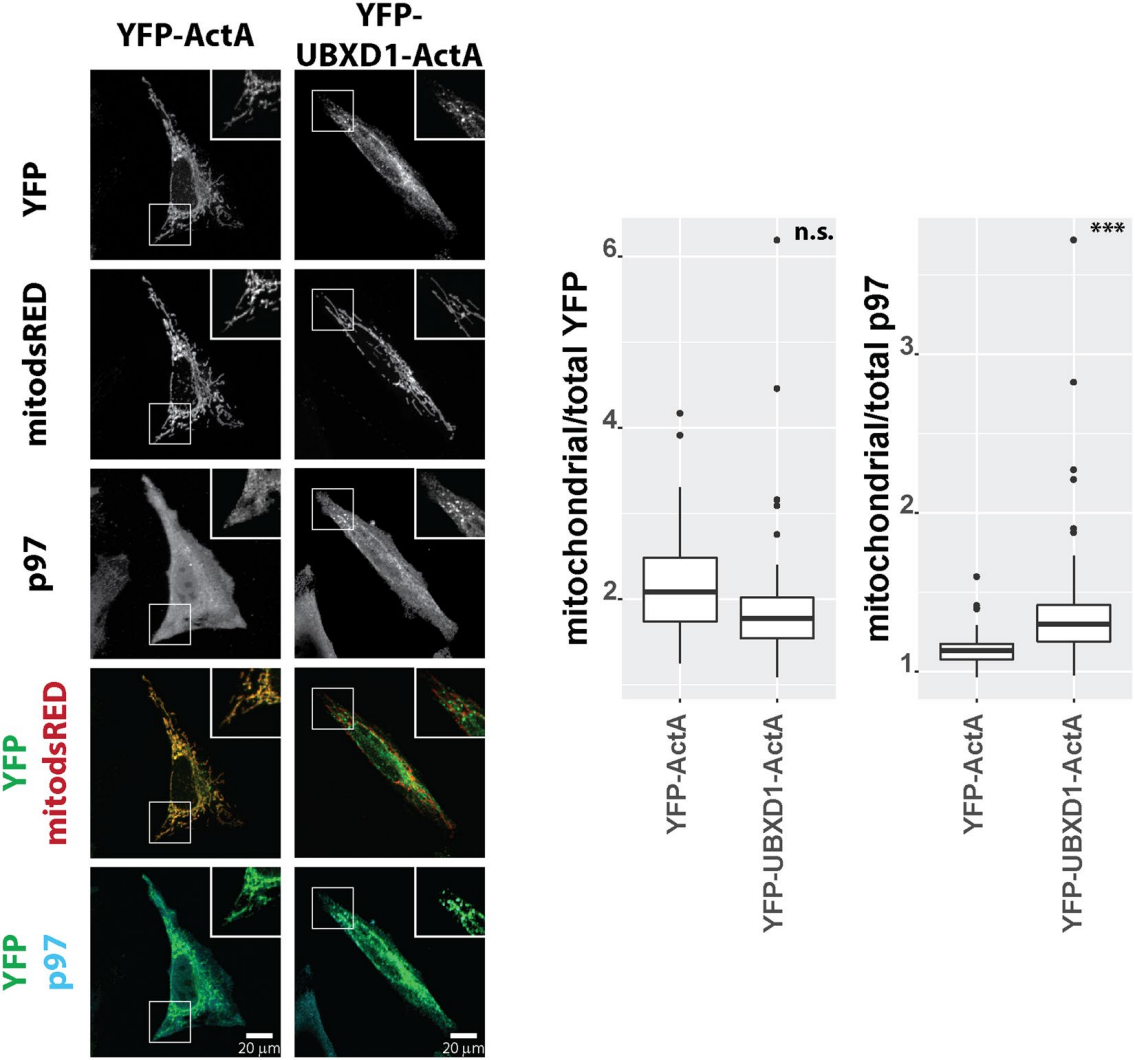
HeLa cells transfected with expression plasmids for mitochondria-targeted dsRED (mitodsRED) and YFP-UBXD1 or YFP fused to the mitochondrial membrane targeting signal ActA were fixed, stained using mouse anti-p97 antibodies, and analyzed by confocal microscopy. Shown are representative images out of three independent experiments. The box plots represent three independent experiments with at least 15 cells/experiment/condition. Statistical significance was assessed by by Student’s t-test. ***denotes p < 0.001, n.s. – no significant difference.

To assess whether UBXD1 alone is sufficient to cause translocation of p97 to mitochondria, we constitutively targeted full-length YFP-tagged UBXD1 or YFP as control to mitochondria through addition of the outer mitochondrial membrane tail anchor ActA. ActA tail targeted YFP-UBXD1 as efficiently as YFP (mitochondrial/total YFP – YFP-ActA: 2.09 +/− 0.56, YFP-UBXD1-ActA: 1.77 +/− 0.35) to mitochondria in the absence of Parkin expression and CCCP treatment (Fig. 9). Whereas endogenous p97 significantly redistributed to mitochondria in cells expressing YFP-UBXD1-ActA, p97 did not show this localization pattern in cells expressing YFP-ActA (mitochondrial/total p97, YFP-ActA: 1.13 +/− 0.08, YFP-UBXD1-ActA: 1.3 +/− 0.17, p = 0.0003). This observation confirms that mitochondria-localized UBXD1 is sufficient for mitochondrial p97 redistribution, without the need for additional factors or signals.

**Figure 10:**
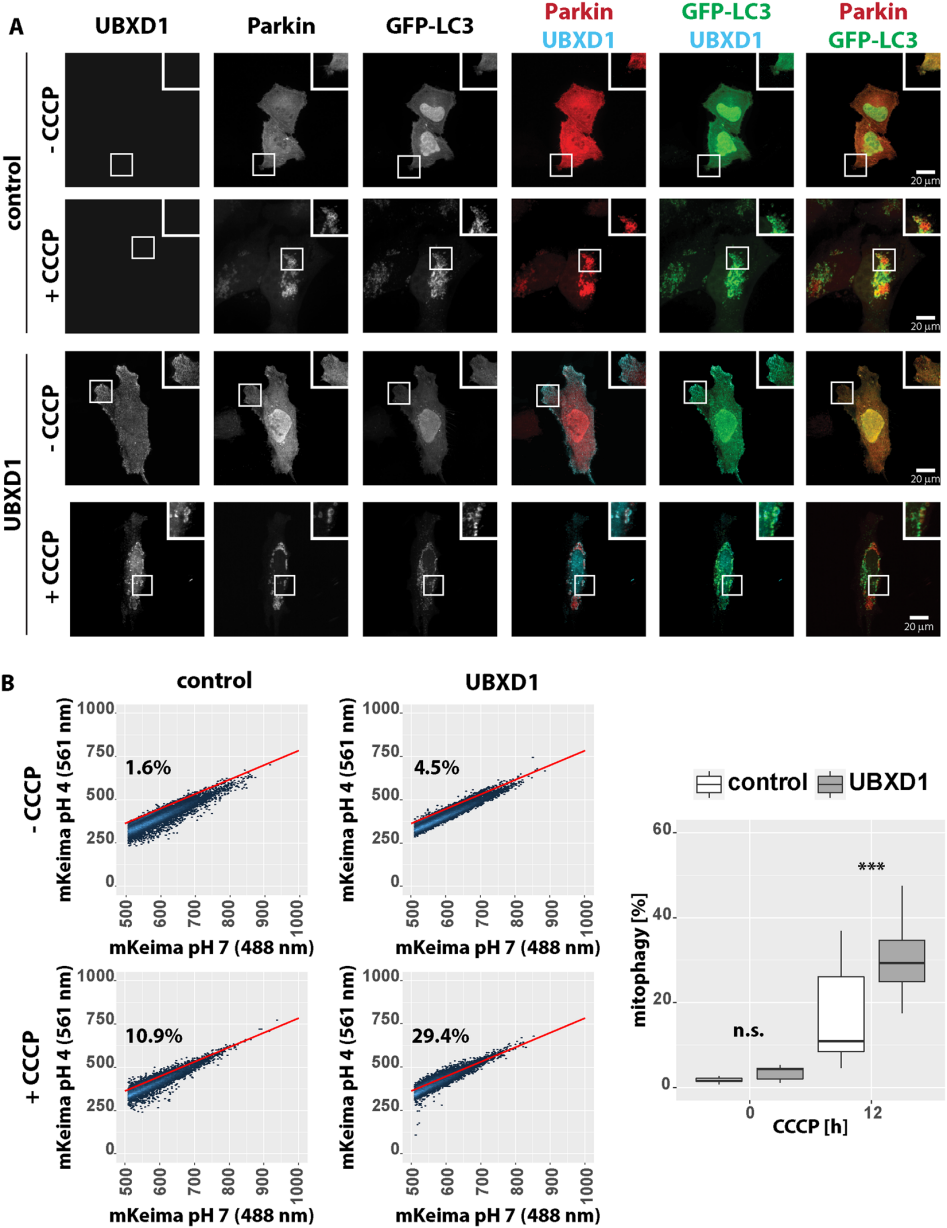
UBXD1 promotes mitophagy. (**A**) HeLa cells transfected with expression plasmids for FLAG-UBXD1 or vector control, mcherry-Parkin and GFP-LC3 were fixed, stained using mouse anti-FLAG antibodies, and analyzed by confocal microscopy. (**B**) HeLa cells transfected with expression plasmids for UBXD1 or vector control and mKeima-T2A-Parkin-myc3 were treated for 12 hours with CCCP or left untreated and analyzed by flow cytometry. Shown are representative density plots (left panels). The box plot represents 6 independent experiments with in total 11 technical replicates. Statistical significance was assessed by ANOVA followed by Student’s t-test using Bonferroni correction to account for multiple comparisons. *** marks p-values < 0.001, n.s. – no significant difference.

A role for p97 in the induction of mitophagy was previously described by Tanaka and co-workers^11^. Thus, recruitment of p97 via UBXD1 might be a critical step for mitophagy onset or progression. To address this question, we studied mitophagic induction and flux in cells ectopically expressing UBXD1. To this end, HeLa cells co-expressing FLAG-UBXD1 and GFP-LC3 in the presence of mcherry-Parkin were treated with CCCP for 6 hours and autophagic vesicle formation was observed. As shown in Fig. 10A, GFP-LC3 positive vesicle density near mitochondria was strongly increased in FLAG-UBXD1 expressing cells compared to controls. Similarly, expression of FLAG-UBXD1 greatly enhanced the formation of GFP-LC3 vesicles near mitochondria upon oligomycin/antimycin treatment compared to vector controls (Figure S6). These observations are indicative of UBXD1 acting pro-mitophagic. To further analyze the impact of UBXD1 on mitophagy, mitophagic flux in cells ectopically expressing FLAG-UBXD1 was measured. HeLa cells co-transfected with expression constructs for the pH-sensitive, mitochondria-targeted reporter mKeima fused to Parkin via the ribosome-cleaved T2A peptide (mKeima-T2A-Parkin-myc3) and UBXD1 or vector control were treated for 12 hours with CCCP to induce mitophagy and analyzed flow cytometrically. The fluorescent reporter mKeima is excitable at 488 nm at pH 7 (inside mitochondria) and 561 nm at pH 4 as encountered inside autolysosomes. Thus, the ratio mKeima_561nm_/mKeima_488nm_ is a measure for mitophagy^23^. In line with the observation of increased GFP-LC3 positive vesicle formation (Fig. 10A), ectopic expression of FLAG-UBXD1 significantly increased (p = 0.0029) mitophagy following addition of CCCP. While 16.8 +/− 11.6% of CCCP treated control cells displayed mitophagy, CCCP treatment triggered mitophagy in 30.8 +/− 9.6% of FLAG-UBXD1-expressing cells (Fig. 10B). Expression of FLAG-UBXD1 in the absence of CCCP treatment did not result in significant induction of mitophagy.

**Figure 11:**
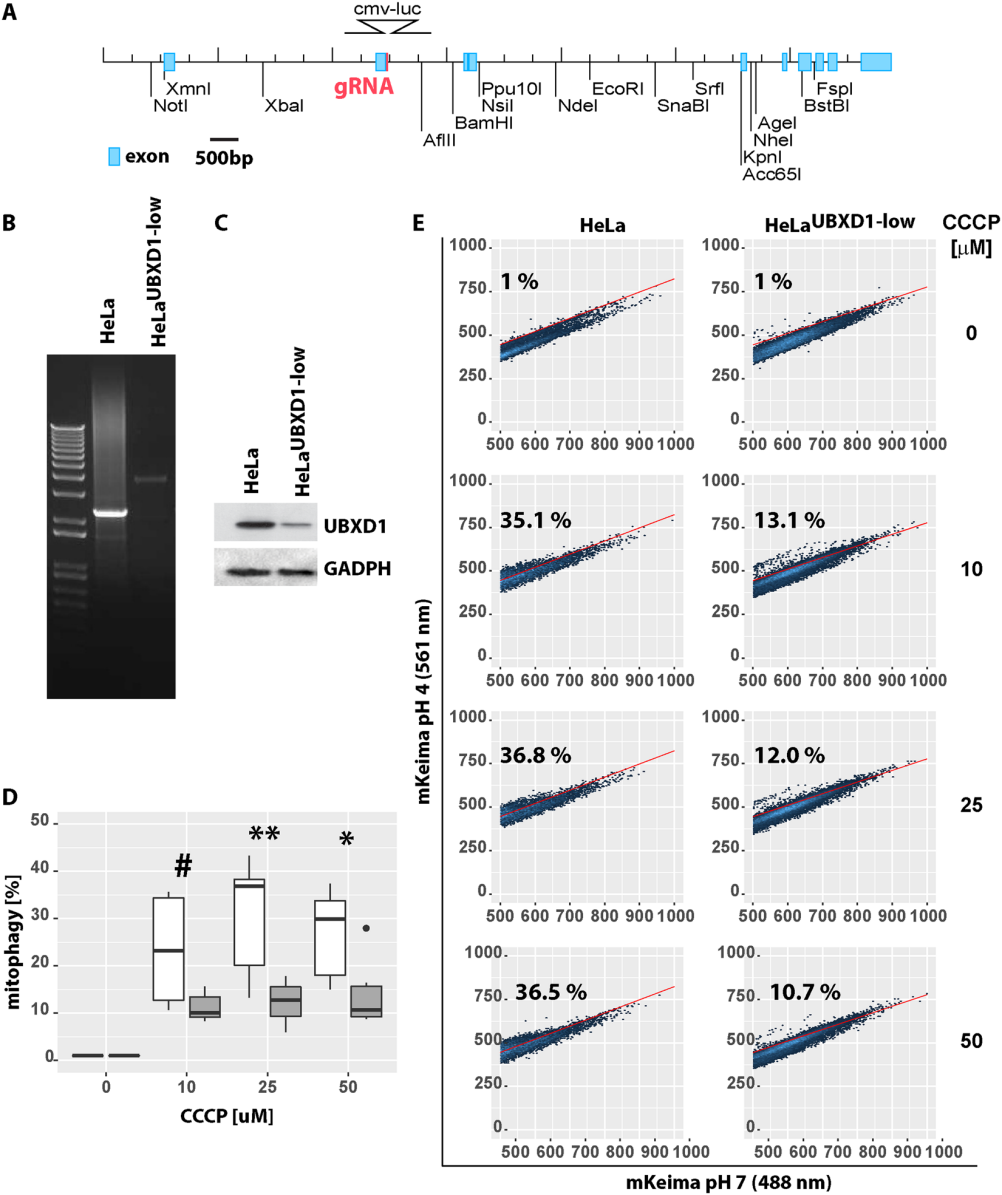
UBXD1 levels influence mitophagic flux. (**A**) Using CRISPR/Cas9, UBXD1 was targeted in HeLa cells and several alleles of UBXD1 were replaced with a reporter cassette coding for secreted Gaussia luciferase. Shown are schematics of CRISPR/Cas9 strategy, (**B**) PCR analysis of reporter integration, (**C**) and levels of UBXD1 in HeLa and HeLa^UBXD1-low^ detected using mouse anti-UBXD1 antibodies. (**D**) HeLa cells HeLa^UBXD1-low^ transfected with mKeima-T2A-Parkin-myc3 were treated for 12 hours with 10, 25 or 50 µM CCCP or left untreated as controls and were analyzed by flow cytometry. The box plot represents 5 independent experiments. (**E**) Shown are representative density plots of the analysis shown in D. Statistical significance was assessed by ANOVA followed by Student’s t-test using correction to account for multiple comparisons according to Holm. * marks p-value < 0.05, **p-value < 0.01, n.s. – no significant difference.

To further assess the role of UBXD1 in modulating mitophagic flux, HeLa cells with diminished levels of UBXD1 were generated. To this end, HeLa cells were transfected with expression constructs for *S. pyogenes* Cas9, an UBXD1 specific gRNA under control of the human U6 promoter and a plasmid with secreted Gaussia luciferase reporter gene flanked by UBXD1 sequences (Fig. 11A) and were single cloned. Following screening for luciferase activity (data not shown) and PCR testing for reporter construct integration (Fig. 11B), UBXD1 levels were analyzed by Western blotting (Fig. 11C). While we were unable to identify cell lines lacking UBXD1 protein, considerable (about 80%) reduction of UBXD1 protein levels were achieved in HeLa^UBXD1-low^ cells. Next, HeLa and HeLa^UBXD1-low^ cells were transfected with mKeima-T2A-Parkin-myc3 and treated with 10, 25 or 50 µM CCCP for 12 hours before flow cytometric analysis. This treatment did not induce cytochrome *c* release in HeLa^UBXD1-low^ cells (Figure S2B). As shown in Fig. 11D and E, diminished levels of UBXD1 resulted in blunted mitophagic flux compared to wildtype control cells. While treatment of wildtype HeLa cells with 10, 25, or 50 µM CCCP resulted in 23.2 +/− 16.3, 36.8 +/− 9.6 and 29.9 +/− 11.1% mitophagic cells, respectively, the percentage of mitophagic HeLa^UBXD1-low^ cells was significantly reduced by at least 50% (10 µM: 10.1 +/− 2.7, 25 µM: 12.8 +/− 5.8, 50 µM 10.7 +/− 2.8%).

## Discussion

Mitochondrial fidelity is maintained by a multitude of interrelated mechanisms working together to uphold morphology, promote repair, but also to remove damaged organelles. Ubiquitination is one recurrent theme connecting these different mechanisms. For OMMAD, mitochondrial proteins are marked by the mitochondrial ubiquitin ligases membrane associated RING-CH 5 (MARCH5)^24–26^ and RNF185^27^ and are retrotranslocated to the cytosol by p97^13,28–30^. Similarly, mitochondrial morphology is regulated by ubiquitin ligases mahogunin Ring Finger-1 (MGRN1)^31^, and MARCH5^32–34^ with the help of p97. Ubiquitination mediated by the ubiquitin ligase Parkin is also postulated to be an important component of mitophagic quality control, involved in constraining, initiating as well as executing mitophagy. In the absence of mitophagy-inducing damage, the Parkin activator PINK1 is degraded by the ubiquitin-proteasome system following export from healthy mitochondria. However, after stabilization of PINK1 on damaged mitochondria, Parkin is recruited, activated by phosphorylation and causes poly-ubiquitination of numerous proteins, significantly resculpting the outer mitochondrial membrane protein landscape^35^. Once activated, Parkin is an unconstrained ubiquitin ligase and forms different poly-ubiquitin chains including chains of the K6, K11, K27, K48 and K63 type on a multitude of mitochondrial proteins^36^. Such attachment of different ubiquitin modifications to target proteins requires recognition and correct processing of these marks. Here, ubiquitin-processing proteins such as p97 and its many co-factors come into play. In the context of mitophagy, p97 was shown to promote mitophagy by assisting in the proteasomal degradation of mitofusins^11^. Beyond mitophagy, p97 is strongly linked to autophagic processes where it is essential for autophagosome formation^37^, the promotion of lysophagy^20^, as well as ER-related autophagy^38^. P97 activity is governed by binding to its many cofactors, with different cofactors tasking p97 with different functions^39^. For example, UBXD1 was shown to target p97 to endoplasmic reticulum (ER) associated degradation^40^, endolysosomal sorting^19^, autophagic degradation of lysosomes^20^, and, most recently, to OMMAD^21^. Our findings now strongly link UBXD1 and in extension p97 to mitophagy. Our data are consistent with UBXD1 recognizing depolarized mitochondria undergoing Parkin-dependent mitophagy and facilitating p97 recruitment. Recognition of mitophagic mitochondria depends exclusively on the UBX domain contained in UBXD1, as evidenced by the complete lack of mitochondrial translocation of UBXD1 missing its UBX domain. Of note, the UBX domain does not to seem contribute to p97 binding, although the UBX domain is generally considered to be a p97 interaction motif. The mitochondrial signal recognized by the UBX domain of UBXD1 remains unknown; however, mitochondrial translocation of UBXD1 critically depends on Parkin activity, as UBXD1 failed to translocate to mitochondria in the absence of Parkin. In support of the observed Parkin-dependence, UBXD1 did not translocate during DFP-induced, Parkin-independent mitophagy.

In line with previous reports^16,41^, the N-terminal VIM and PUB domain are both sufficient to bind p97 and promote mitochondrial recruitment. Upon ectopic expression, both domains are individually capable of recruiting p97 to mitochondria under mitophagic conditions although quantitative yeast two-hybrid suggested additive binding strength. This observation points to additional functions of the bipartite p97 binding motif in UBXD1; indeed, it was proposed that UBXD1 contacts p97 at N- as well as C-terminal locations, thereby modulating accessibility of other p97 cofactors^16^. Therefore, it is likely that the bipartite p97 interaction motif consisting of the VIM and PUB domain serves similar functions during mitophagy and not only recruits p97 to mitochondria but might also adjust cofactor binding of p97 under mitophagic conditions.

Interestingly, unlike mitochondrial translocation of UBXD1, UBXD1 binding to p97 occurred independently of mitophagy. Targeting UBXD1 to mitochondria by adding the ActA tail was sufficient to induce mitochondrial recruitment of p97, and did not require mitophagic signals. This observation supports the notion that the UBX domain of UBXD1 is sufficient to recognize mitophagic mitochondria and that p97 recruitment does not rely on additional signals.

As for the role of UBXD1-mediated, mitochondrial recruitment of p97, our data supports a pro-mitophagic role for UBXD1. The formation of LC3-containing vesicles near mitochondria, the significant increase in mitophagic flux after ectopic expression of UBXD1, and the significant decrease of mitophagy in HeLa^UBXD1-low^ cells supports this notion. While it remains unclear how UBXD1 causes the observed increase in mitophagic flux, it is likely that removal of certain mitochondrial proteins by UBXD1-p97 complexes is involved; in a similar scenario, p97-mediated removal of mitofusins was shown to promote mitophagy^11^. Additionally, UBXD1 in concert with p97 and other cofactors was recently shown to promote the removal of K48-linked ubiquitin from ruptured lysosomes thereby promoting lysophagy^20^. A similar mechanism might be at work during mitophagy. Parkin is known to form various types of poly-ubiquitin among them K48 and K63 linked chains. Thus, removal of K48-ubiquitin tagged client proteins by UBXD1-p97 complexes might increase K63-ubiquitin chains on mitochondria in turn promoting p62 recruitment and autolysosome formation. However, it remains to be determined whether such a mechanism is at work during mitophagy.

UBXD1 and p97-mediated quality control is of clinical relevance. Mutations in p97 causing myopathy and neurodegeneration were shown to hamper interaction with UBXD1, resulting in impaired degradation and subsequent accumulation of p97 client proteins^19^. Also, UBXD1 was recently connected to the degradation of mitochondrial MCL1 in the context of an *in vitro* model of Huntington’s disease^21^. Furthermore, impaired, autophagic removal of ruptured endolysosomes, e.g. following uptake of neurotoxic tau fibrils, was reported to be UBXD1-p97 mediated^20^. Since impaired mitophagy is strongly linked to neurodegenerative diseases, it is tempting to speculate that, due to altered binding of mutant p97 to UBXD1, p97-linked myopathy and neurodegeneration might not only be a consequence of disrupted lysophagy, but might also result from impaired mitophagic clearance of damaged mitochondria.

Taken together, UBXD1 as mitochondrial recruitment factor for p97 further connects the ubiquitin-proteasome system to Parkin-dependent mitophagy and underlines the close cooperation between the different mechanisms involved in mitochondrial maintenance.

## Material and Methods

### Cell culture and treatments

Hela cells were cultured in Dulbecco’s modified Eagle’s medium (DMEM) supplemented with 2 mM L-glutamine, 1 mM sodium pyruvate, and 10% fetal bovine serum (Sigma-Aldrich). Cells were incubated in a humidified atmosphere at 5% CO_2_ and 37 °C. HeLa cells were transfected using Fugene (Promega) or PEI MAX 40000 (24765, PolySciences, Inc., Warrington) according to the manufacturer’s recommendations. HeLa cells were treated with 0 to 50 µM carbonyl cyanide *m*-chlorophenyl hydrazine (CCCP – Sigma-Aldrich), 10 µM oligomycin (Enzo Lifesciences), 1 µM antimycin (Enzo Lifesciences), 100 nM bafilomycin (Sigma-Aldrich) or 1 mM 1,2-dimethyl-3-hydroxy-4(1 H)-pyridon (deferiprone - DFP for the indicated time (Sigma-Aldrich).

### Immunofluorescence and confocal microscopy

For immunofluorescence, cells were fixed with 4% paraformaldehyde (Pierce) in PBS, permeabilized with 0.15% Triton-X100 (Sigma) in PBS, blocked with 10% BSA (Carl Roth AG) in PBS, and incubated with primary antibody overnight at 4 °C and secondary antibody for 2 hours at RT in 1% BSA/PBS. As primary antibodies rabbit anti-FLAG (Thermo Fisher Scientific, PA1-984B), mouse anti-FLAG (Sigma, F1804), mouse anti-myc (Sigma, M5546) as well as mouse anti-p97 (Thermo Fisher Scientific, MA3-004) were employed. As secondary antibodies anti-mouse and anti-rabbit antibodies labelled with Alexa680 or Alexa564 (Life Technologies) were used. Cells were mounted in Vectashield H1000 (Vector Labs) and analyzed using a Visitron CSU-W1 Thor confocal microscope.

### Western blotting

To detect UBXD1 protein, protein lysates of HeLa cells were prepared using RIPA buffer, separated on 12%SDS-PAGE, and analyzed using mouse anti-UBXD1 (Abcam, 80659), mouse α-GAPDH (1:3000; Santa Cruz #sc-32233), anti-FLAG (1:1000, Sigma F1804), anti-myc (1:2000, Sigma M5546) and anti-mouse HRP (1:20000; ThermoFisher) antibodies. Yeast cells were lysed using acid-washed glass beads (Sigma) in yeast protein buffer (100 mM NaCl, 50 mM NaF, 5 mM EDTA, 0.1% IgepalCA-630, 50 mM Tris/Cl pH7.5) supplemented with complete Ultra protease inhibitor mix (Roche).

### DNA constructs

Refer to Table S1 and Table S2 for details on plasmid construction. All DNA constructs were verified by sequencing (Microsynth).

### Yeast two-hybrid

The MatchMaker Yeast Two-Hybrid Gold system (Clontech) was used according to manufacturer’s recommendations. Yeast cells were grown on synthetic media lacking leucine and tryptophan as control and on synthetic media lacking leucine, tryptophan, adenine, histidine and containing X-alpha-Gal (Sigma) and Aureobasidin A to select for two hybrid interaction.

### Quantitative mitophagy assay

Cells transfected with expression construct for mKeima and Parkin (mKeima-T2A-Parkin-myc3) as well as UBXD1 or control vector were treated with CCCP or left untreated. Cells were harvested and mKeima fluorescence at 488 nm and 561 nm excitation was measured by flow cytometry (BC Cytoflex). Cells were gated to exclude debris (FlowJo) and cells not expressing mKeima (488 nm). To quantify mitophagy, cells expressing mKeima and transfected with vector control without CCCP treatment were used to establish mKeima_561nm_/mKeima_488nm_ threshold. Using R^42^, a linear model (shown as red line in Fig. 7B) was established based on the mean of the 99^th^ percentile mKeima_561nm_ for each mKeima_488nm_ value using the R quantreg package^43^. Cells above the mKeima_561nm_/mKeima_488nm_ threshold were counted to determine percentage of cells with above control mitophagy levels.

### Statistical analysis

Statistical analysis was performed using R as indicated. All experiments were performed independently at least three times. Statistical significance was assessed by ANOVA with posthoc two-tailed Student’s t-test using Bonferroni or Holm adjustment to account for multiple comparisons. P-values > 0.05 are marked with n.s., p < 0.05 with *p < 0.01 with **p < 0.001 with ***.

## Electronic supplementary material

Supplementary Information

## Data Availability

Materials, data and associated protocols are available without restriction upon request.
