# Supplementary material for: UBXD1 is a mitochondrial recruitment factor for p97/VCP and promotes mitophagy
Source: Sci Rep. 2018 Aug 17;8:12415. doi: 10.1038/s41598-018-30963-z (PMC6098094; doi:10.1038/s41598-018-30963-z)
Supplement: Supplementary file 1 — Supplementary Information [file 41598_2018_30963_MOESM1_ESM.docx]

**Supplemental Data**

**UBXD1 is a mitochondrial recruitment factor for p97/VCP and promotes mitophagy**

Ana Catarina Pinho Ferreira Bento^1^, Claudia C. Bippes^1^, Corina Kohler^1^, Charles Hemion^1^, Stephan Frank^2^, Albert Neutzner^1,3,§^

^1^Department of Biomedicine, University Hospital Basel and University of Basel, Switzerland

^2^Department of Pathology, University Hospital Basel, University of Basel, Switzerland

^3^Department of Ophthalmology, University Hospital Basel, University of Basel, Basel, Switzerland

^§^ address correspondence to: Albert Neutzner, University Basel, Department of Biomedicine, Hebelstrasse 20, 4031 Basel, Switzerland. Email: albert.neutzner@unibas.ch

# phone: +41 61 265 3534

**
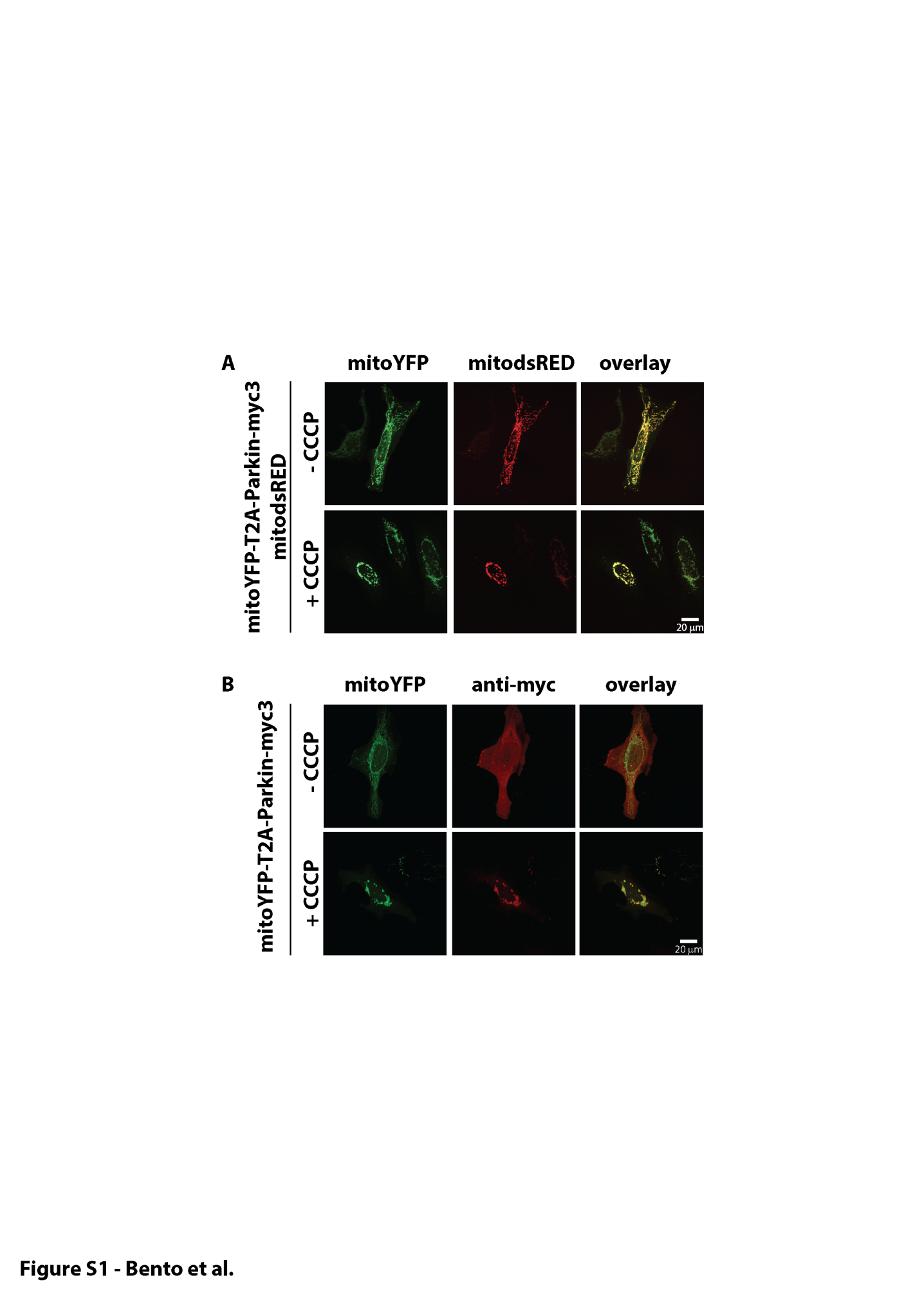
**

**Figure S1:** Combined expression of mitochondria-targeted YFP and Parkin. (A) HeLa cells transfected with an expression plasmid for mitodsRED and a fusion protein between mitoYFP and Parkin-myc3 separated with the cotranslationally-cleaved T2A peptide were analyzed by confocal microscopy after treatment with CCCP for 6 hours or no treatment as control. Note that mitoYFP and mitodsRED colocalize and that both markers show the typical CCCP-induced mitochondrial fragmentation. (B) HeLa cells transfected with mitoYFP-T2A-Parkin-myc3 were treated with CCCP for 6 hours or left untreated, fixed and stained using mouse anti-myc antibodies. Please note the lack of co-localization between mitoYFP and Parkin-myc3 in the absence of CCCP and the overlap of both mitoYFP and Parkin in the presence of CCCP, confirming intended cotranslational processing of mitoYFP-T2A-Parkin-myc3.

**
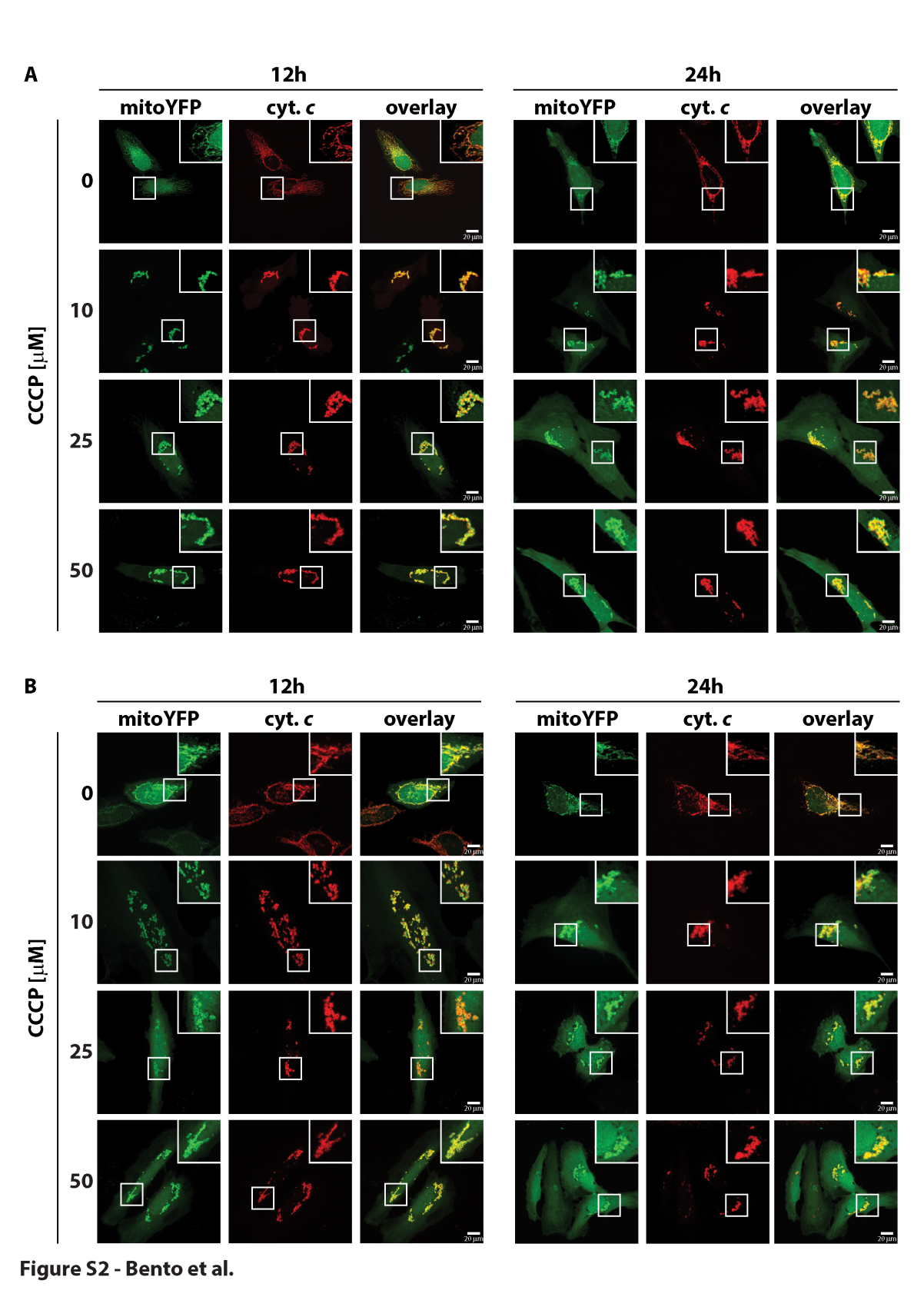
**

**Figure S2:** CCCP treatment does not induce apoptosis in Hela and HeLa^UBXD1-low^ cells. HeLa (A) or HeLa^UBXD1-low^ cells (B) transfected with mitoYPF-T2A-Parkin-3xmyc were treated for 12 or 24 hours with 0, 10, 25, or 50 µM CCCP, fixed, stained using anti-cytochrome *c* antibodies and analyzed by fluorescence microscopy. Under these conditions, no release of cytochrome *c* was observed. Shown are representative images of three independent experiments.

**
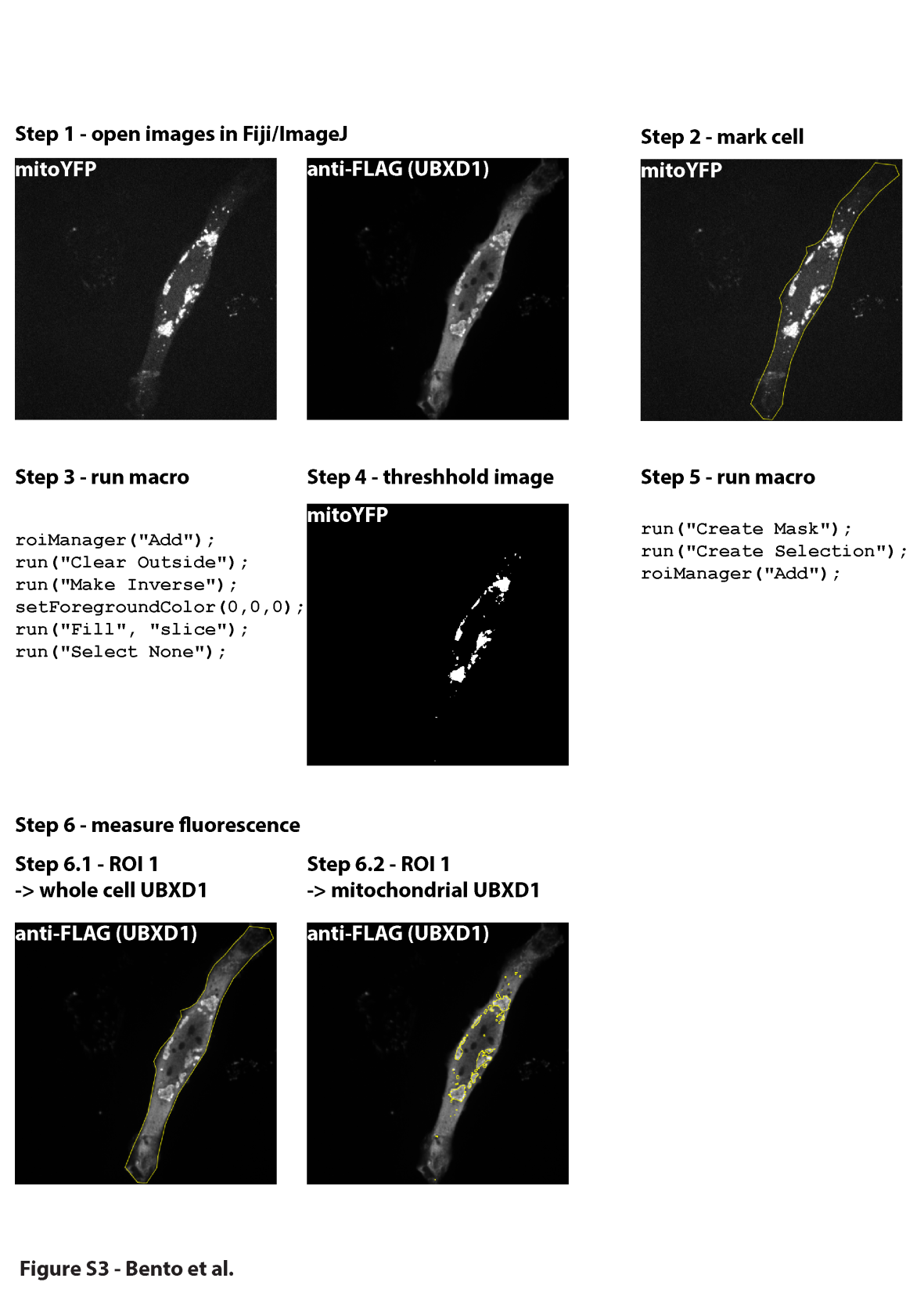
**

**Figure S3:** Image analysis work flow for quantification of mitochondrial translocation. Image analysis was performed using Fiji. ^1^ After opening corresponding images (Step 1) of mitoYFP (mitochondria) and anti-FLAG staining (UBXD1), a region of interest (ROI) was drawn on the mitoYFP image to mark the cell of interest (Step 2). Using the macro shown in Step 3, this ROI (ROI 1 = cell area) was transferred to the ROI manager and the cell was isolated. Using the thresholding function (Step 4), a binary image was generated selecting mitochondria. Using the macro in Step 5, a mask and selection was generated and added as ROI to the ROI manager (ROI 2 = mitochondrial area). Using ROI1 and ROI2, median fluorescence was measured reflecting cellular UBXD1 (ROI1) and its mitochondrial subset (ROI2). Please note to avoid bias from vastly different protein expression levels, cells with similar (by eye) expression levels of UBXD1 or UBXD1 mutants were imaged.


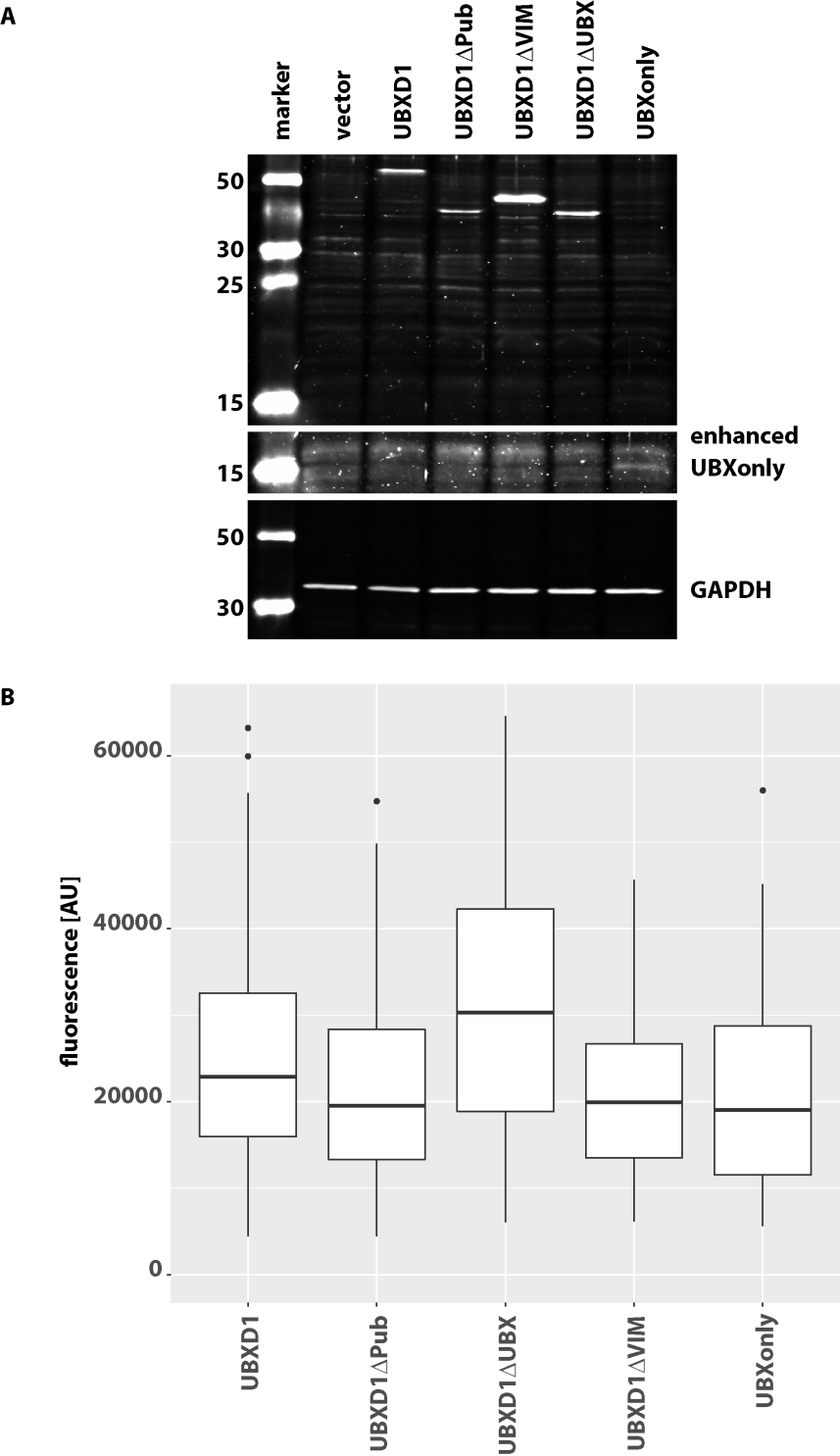


**Figure S4:** Expression levels analysis of UBXD1 variants. (A) Protein lysates of HeLa cells transfected with expression constructs for FLAG-UBXD1, FLAG-UBXD1ΔPUB, FLAG-UBXD1ΔVIM, FLAG-UBXD1ΔUBX, or FLAG-UBXonly were analyzed by Western blotting (15 % SDS-PAGE) using anti-FLAG and anti-GAPDH antibodies. Note that UBXonly is expressed at lower levels compared to the other UBXD1 variants (second panel is enhanced to show expression of UBXonly). (B) Cells with visually similar expression of UBXD1 variants including UBXonly were imaged for mitochondrial translocation analysis. The boxplot depicts the total cellular fluorescence representing UBXD1 variant expression in cells used for single cell analysis presented in Figure 4. Note that expression levels of all UBXD1 variants are comparable between the analyzed cell groups.

**
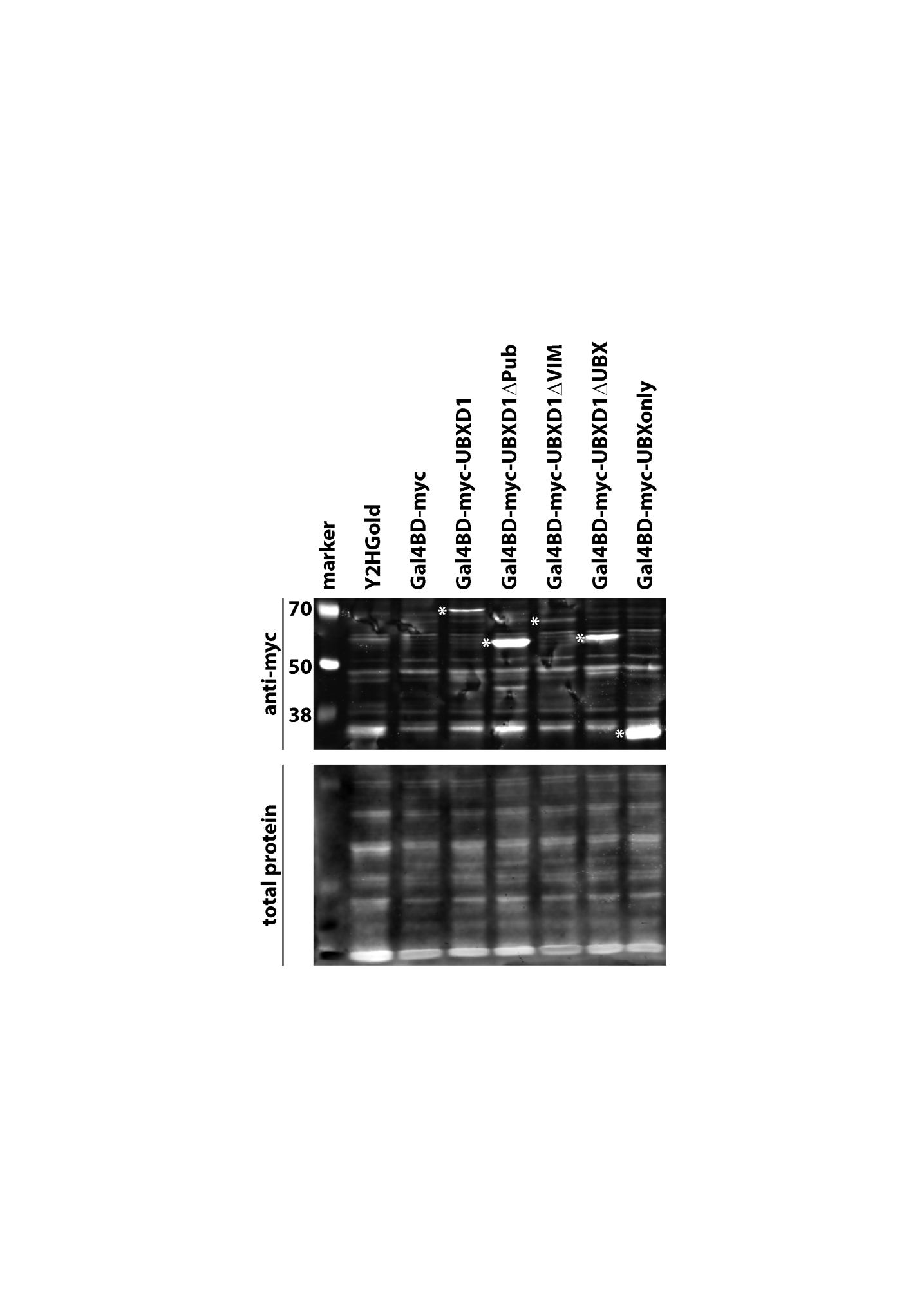
**

**Figure S5:** Testing expression of bait constructs used in yeast two hybrid analyses. Protein lysates of cells of yeast strain Y2HGold and Y2HGold transformed with the prey plasmid Gal4AD-p97 and the indicated bait plasmids were analyzed by Western blotting (9 % SDS-PAGE) using anti-myc antibodies. Detection of total protein using FastGreen FCF served as loading control.


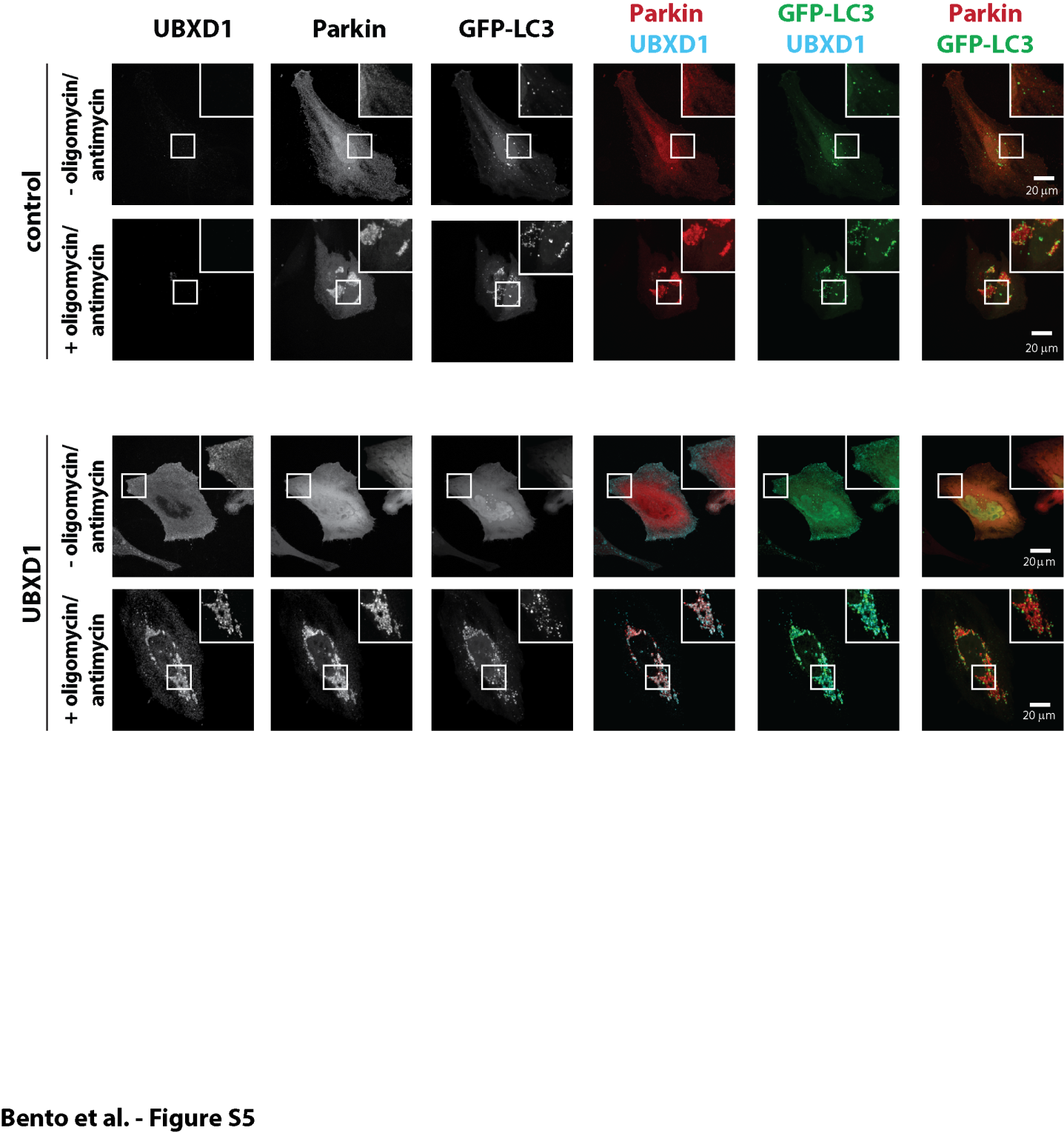


**Figure S6:** UBXD1 increases formation of GFP-LC3 vesicles following treatment with oligomycin/antimycin. HeLa cells transfected with expression plasmid for mcherry-Parkin, GFP-LC3 and FLAG-UBXD1 or vector control were treated with 10 µM oligomycin and 1 µM antimycin for 6 hours and analyzed by confocal microscopy. Shown are representative images of two independent experiments.

| **Name** | **Description** | **Vector** | **Insert** |
| --- | --- | --- | --- |
| pAN940 | YFP-Parkin | gift from Richard Youle |  |
| pAN941 | mcherry-Parkin | gift from Richard Youle |  |
| pGBKT7 | GAL4BD (bait) | Matchmaker (Clontech) |  |
| pGADT7 | GAL4AD (prey) | Matchmaker (Clontech) |  |
| pAN3198 | mKeima | addgene #56018 | mKeima |
| pAN2741 | FLAG-UBXD1 | pcDNA3.1+-DYK | UBXD1 (Genscript: OHu09321) |
| pAN2861 | YFP-UBXD1 | EYFP-C1 (Clontech) | pAN2741 PCR: OAN2020/2021 |
| pAN2862 | FLAG-UBXD1ΔPUB | pcDNA3.1 **Eco*RI, *Hin*dIII | pAN2741 PCR: OAN2022/2025 **Eco*RI, *Hin*dIII |
| pAN2863 | FLAG-UBXD1ΔUBX | pcDNA3.1 **Eco*RI, *Xho*I | pAN2741 PCR: OAN2022/2024 **Eco*RI, *Xho*I |
| pAN3104 | FLAG-UBXD1ΔVIM | pAN2741**Hin*dIII, *Eco*RV | pAN2741 PCR: OAN2412/14 **Hin*dIII, *Eco*RV |
| pAN3105 | FLAG-UBXonly | pAN2741**Eco*RV, *Hin*dIII | pAN2741 PCR: OAN2413/14 **Hin*dIII, *Eco*RV |
| pAN3307 | FLAG-VIMonly | pAN2855 *EcoRI/XhoI | pAN2741 PCR: OAN2022/2675 *EcoRI/XhoI |
| pAN3080 | YFP-UBXD1-ActA | pAN2861 **Xho*I, *Eco*RI | pAN2861 PCR: OAN2333/2334 **Xho*I, *Eco*RI |
| pAN3187 | YFP-ActA | pcDNA3 **Bam*HI, *Hin*dIII | YFP-C1 PCR: OAN2504/2505 **Bam*HI, *Hin*dIII |
| pAN3090 | GAL4BD-UBXD1 | pGBTK7 **Nde*I, *Bam*HI | pAN2861 PCR: OAN2398/9 **Nde*I, *Bam*HI |
| pAN3091 | GAL4BD-UBXD1ΔPUB | pGBTK7 **Nde*I, *Not*I | pAN2862 PCR: OAN2400/01 **Nde*I,*Not*I |
| pAN3092 | GAL4BD-UBXD1ΔUBX | pGBTK7 **Nde*I, *Bam*HI | pAN2863 PCR : OAN2400/02 **Nde*I, *Bam*HI |
| pAN3093 | GAL4BD-UBXD1ΔVIM | pGBTK7 **Nde*I, *Bam*HI | pAN2861 PCR: OAN2403/2399 **Nde*I, *Bam*HI |
| pAN3094 | GAL4BD-UBXonly | pGBTK7 **Nde*I, *Bam*HI | pAN2861 PCR: OAN2404/2399 **Nde*I, *Bam*HI |
| pAN3103 | mitoYFP-T2A-Parkin-myc3 | pAN940**Nhe*I, *Hin*dIII | mitoYFP PCR: OAN2415/2416 **Nhe*I, *Hin*dIII |
| pAN3216 | mKeima-T2A-Parkin-m<c3 | pAN940 **Nhe*I, *Hin*dIII | pAN3198: OAN2518/2519 **Nhe*I, *Hin*dIII |
| pX459^2^ | CRISRP/Cas9 | pSpCas9(BB)-2A-Puro (PX459) gift from Feng Zhang (Addgene # 48139) | |
| pAN3046 | UBXD1-CRISPR/Cas9 | pX459 *BbsI | Annealed OAN2272/2273 |

Table S1: DNA constructs used in this study.

| **Name** | **Sequence** |
| --- | --- |
| OAN2020 | TCATCTCTCGAGCTATGAAGAAATTCTTTCAGGAGTTCAAG |
| OAN2021 | TGTACTAAGCTTTCACAAGAGCTTCTCGATGGCT |
| OAN2022 | TCATCTGAATTCATGGATTACAAGGATGACGACGA |
| OAN2024 | TCTATCCTCGAGTCACCGCTTGATCTCCTCTGCTGT |
| OAN2025 | TGTACTGGATCCTGAGCCACCTGAGCCACCGTTGAACGTGTAGATCTTCATGAT |
| OAN2333 | GTCCGGACTCAGATCTCGAG |
| OAN2334 | AGATGAGAATTCTCAATTGTTTTTTCTCAGCTGAATAATTTTGATAAAGGCGCCCAGGGA  GAACACGCCAATAGCCAGCATGGCGAGAATCAGCAAGAGCTTCTCGATGGCTG |
| OAN2398 | AGATGACATATGAAGAAATTCTTTCAGGAGTTCAAGG |
| OAN2399 | TCATCTGGATCCTCACAAGAGCTTCTCGATGGC |
| OAN2400 | AGATGACATATGAAGAAATTCTTTCAGGAGTTCAAGGC |
| OAN2401 | TCATCTGCGGCCGCTCACAAGAGCTTCTCGATGGC |
| OAN2402 | TCATCTGGATCCTCACCGCTTGATCTCCTCTG |
| OAN2403 | AGATGACATATGTCGCAGGACACCATCCg |
| OAN2404 | AGATGACATATGCGGGAGCAGAGGCTCAGg |
| OAN2412 | GCTCGTAAGCTTATGGATTACAAGGATGACGACGATAAGTCGCAGGACACCATCCG |
| OAN2413 | GCTCGTAAGCTTATGGATTACAAGGATGACGACGATAAGCGGGAGCAGAGGCTCAGG |
| OAN2414 | TCATCTGATATCTCACAAGAGCTTCTCGATGGC |
| OAN2415 | AGATGAGCTAGCATGTCCGTCCTG |
| OAN2416 | AGATGAAAGCTTTCAGATCTTCTTCAGAGATGAGTTTCTGCTCAGGGCCGGGATTCTCCTCCACGTCACCGCATGTTAGAAGACTTCCTCTGCCCTCCTTGTACAGCTCGTCCATGC |
| OAN2504 | CTTGAAGCTTAGACATGGTGAGCAAGGGC |
| OAN2505 | GGCTGGATCCTCAATTGTTTTTTCTCAGCTGAATAATTTTGATAAAGGCGCCCAGGGAGAACACGCCAATAGCCAGCATGGCGAGAATCAGCTTGTACAGCTCGTCCATG |
| OAN2518 | AGATGAGCTAGCATGTCCGTCCTGACG |
| OAN2519 | AGATGAAAGCTTTCAGATCTTCTTCAGAGATGAGTTTCTGCTCAGGGCCGGGATTCTCCTCCACGTCACCGCATGTTAGAAGACTTCCTCTGCCCTCACCGAGCAAAGAGTGG­ |
| OAN2675 | TCATCT CTCGAG TTA TGTGGGGCCCCAGGC |
| OAN2272 | CACCGCCTTGATGAGCCGCTCCCAA |
| OAN2273 | AAACTTGGGAGCGGCTCATCAAGGC |

Table S2: Sequences of oligonucleotides used in this study. All oligonucleotides were purchased from Sigma.

## Supplemental References

1 Schindelin, J. *et al.* Fiji: an open-source platform for biological-image analysis. *Nat Methods* **9**, 676-682, doi:10.1038/nmeth.2019 (2012).

2 Ran, F. A. *et al.* Genome engineering using the CRISPR-Cas9 system. *Nat Protoc* **8**, 2281-2308, doi:10.1038/nprot.2013.143 (2013).
